# Redox-induced reversible [2 + 2] cycloaddition of an etheno-fused diporphyrin†

**DOI:** 10.1039/d1sc00438g

**Published:** 2021-02-24

**Authors:** Kazuya Miyagawa, Ichiro Hisaki, Norihito Fukui, Hiroshi Shinokubo

**Affiliations:** Department of Molecular and Macromolecular Chemistry, Graduate School of Engineering, Nagoya University Furo-cho, Chikusa-ku Nagoya 464-8603 Japan hshino@chembio.nagoya-u.ac.jp; Graduate School of Engineering Science, Osaka University 1-3 Machikaneyama, Toyonaka Osaka 560-8531 Japan

## Abstract

3,5-Ethenoporphyrin is a π-extended porphyrin containing a fused ethene unit between the *meso*- and β-positions, exhibiting unique contribution of macrocyclic antiaromaticity. We have recently reported that its analogue, etheno-fused diporphyrin, underwent thermal [2 + 2] cycloaddition to furnish X-shaped cyclobutane-linked tetraporphyrins. Here we demonstrate that the cyclobutane-ring formation is dynamically redox-active. Namely, the tetraporphyrin underwent two-step four-electron oxidation to afford two etheno-fused diporphyrin dications. The reduction of the resulting dication regenerated the cyclobutane-linked tetraporphyrin. The dication was sufficiently stable to allow its isolation under ambient conditions. The structure of the dication has been confirmed by ^1^H NMR spectroscopy and X-ray diffraction analysis. Importantly, the simultaneous double C–C bond cleavage in the cyclopropane ring in the tetraporphyrin is exceptional among dynamic redox (dyrex) systems to achieve large structural changes, thus offering new insights for the design of novel redox-active functional organic materials for electrochromic dyes, organic batteries, and organic memories.

## Abstract 2

A four-electron oxidation of an X-shaped tetraporphyrin affords stable etheno-fused diporphyrin dications through double C–C bond cleavage. The reduction of the dication recovers the tetraporphyrin *via* a thermal [2 + 2] cycloaddition.

## Graphical abstract

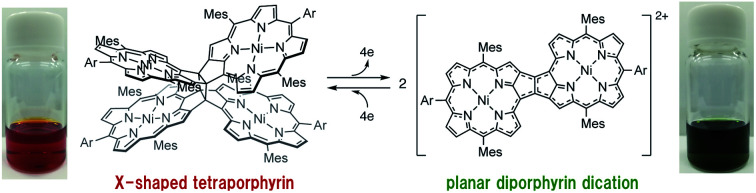

## Introduction

**Fig. 1:**
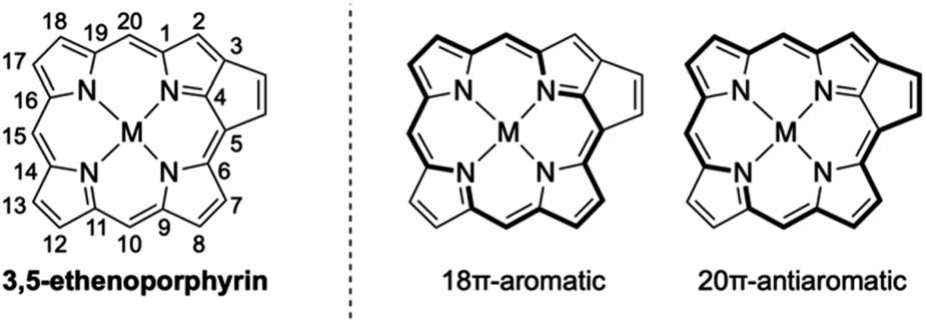
Aromatic and anti-aromatic conjugation circuits of 3,5-ethenoporphyrin.

Porphyrins with extended π-conjugation networks exhibit numerous intriguing properties, such as near-infrared absorption, reversible redox activity, characteristic chemical reactivity, and high single-molecule conductance.^1^ Such porphyrins have attracted considerable attention in various research fields including organic and supramolecular chemistry as well as materials science. 3,5-Ethenoporphyrin is an extraordinary π-extended porphyrin due to the coexistence of 18π-aromaticity and 20π-antiaromaticity in its macrocyclic conjugation (Fig. 1).^2,3^ Consequently, 3,5-ethenoporphyrin exhibits a narrow HOMO–LUMO gap and high reactivity of the fused C–C double bond.

**Fig. 2:**
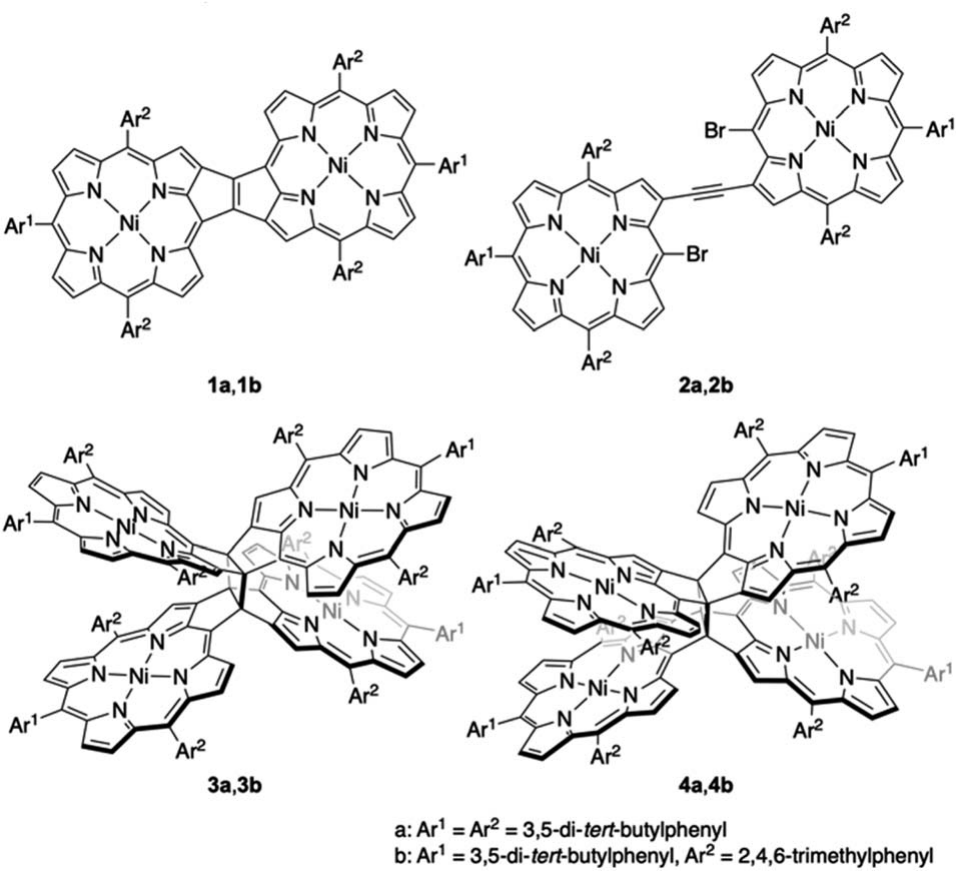
Etheno-fused diporphyrin **1**, ethynylene-linked dibromodiporphyrin **2**, and cyclobutane-linked tetraporphyrins **3** and **4**.

Recently, our research group envisaged the addition of another fused-porphyrin unit to the 3,5-ethenoporphyrin skeleton and attempted the synthesis of etheno-fused diporphyrin **1a***via* the tandem double-cyclization of β,β-ethynylene-linked dibromodiporphyrin **2a** (Fig. 2).^4^ Unexpectedly, we discovered the formation of cyclobutane-linked tetraporphyrins **3a** and **4a**. These two tetraporphyrins were formed through the thermal [2 + 2] cycloaddition reaction of *in situ*-generated **1a**. Due to orbital symmetry, the formation of the cyclobutane in **3a***via* a [2 + 2] cycloaddition is thermally forbidden. Thus, the formation of **3a** and **4a** suggests the involvement of a thermally activated triplet state of **1a** in the thermal [2 + 2] cycloaddition reaction. Indeed, *syn*-tetramer **4a** isomerizes to *anti*-tetramer **3a** upon heating to 160 °C, which implies that the [2 + 2] cycloaddition of **1a** is thermally reversible.

We then decided to conduct further investigations into these cyclobutane-linked tetraporphyrins with a focus on their redox properties. Here, we disclose a reversible [2 + 2] cycloaddition through electron-transfer-induced cyclobutane ring-opening and -closure. This redox-induced reversible C–C-bond formation constitutes a dynamic redox (dyrex) system; such systems have been actively explored on account of their potential importance as electrochromic dyes, organic batteries, and organic memory devices.^5,6^ Importantly, the simultaneous double C–C-bond formation/cleavage observed in the tetraporphyrin system is exceptional among reported dyrex systems.

## Results and discussion

### Synthesis and characterization of cyclobutane-linked tetraporphyrin

**Scheme 1:**
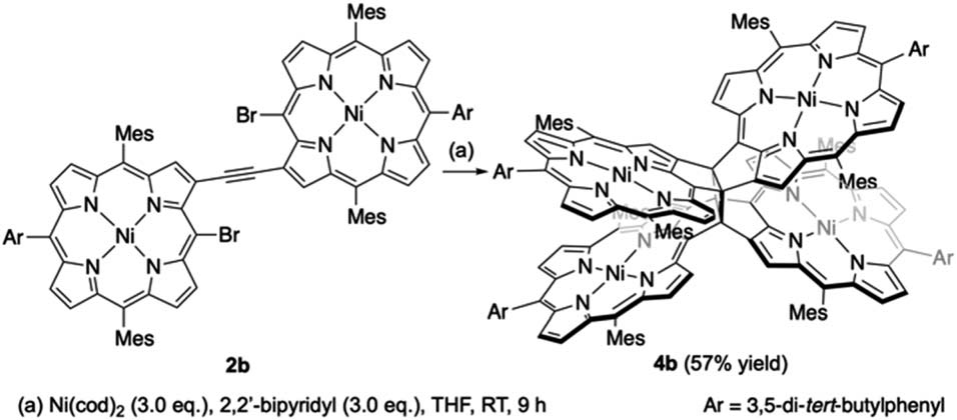
Synthesis of *syn*-tetramer **4b**.

**Fig. 3:**
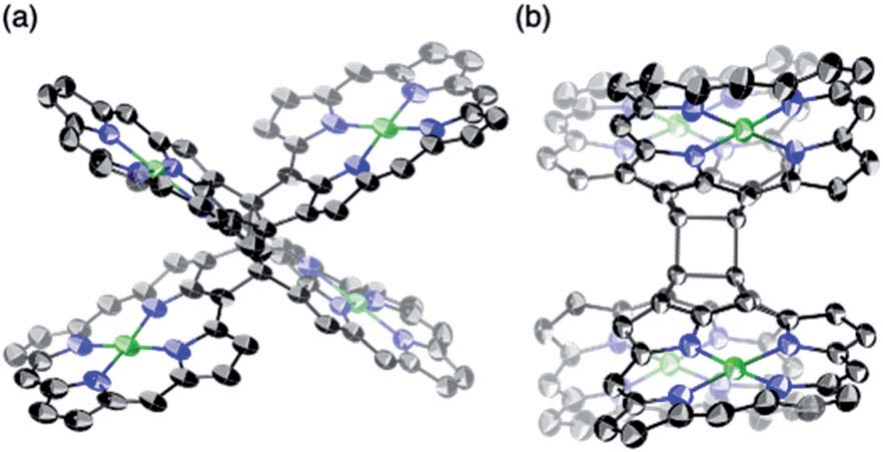
Single-crystal X-ray crystal structure of *syn*-tetramer **4b**. (a) General and (b) side view. Thermal ellipsoids are drawn at 50% probability. Solvent molecules, peripheral aryl groups, and all hydrogen atoms are omitted for clarity.

β,β-Ethynylene-linked dibromodiporphyrin **2b** was prepared according to a slightly modified literature procedure.^4,7^ Precursor **2b** was subjected to a tandem double cyclization with Ni(cod)_2_, which afforded *syn*-tetramer **4b** in 57% yield without the formation of *anti*-tetramer **3b** (Scheme 1). *anti*-Tetramer **3b** was not obtained even when the reaction temperature was increased to 60 °C. The selective formation of **4b** is due to the steric effect by bulky mesityl groups at the *meso*-positions. The structure of *syn*-tetramer **4b** was unambiguously determined by single-crystal X-ray diffraction analysis (Fig. 3).

### Dyrex response of cyclobutane-linked tetraporphyrin

#### Cyclic voltammogram

**Fig. 4:**
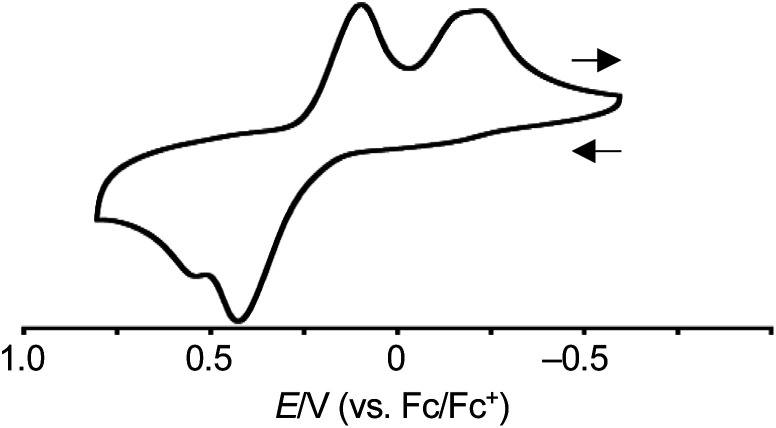
Cyclic voltammogram of **4b**. Solvent: CH_2_Cl_2_; supporting electrolyte: 0.1 M [Bu_4_N][PF_6_]; working electrode: glassy carbon; counter electrode: Pt; reference electrode: Ag/AgNO_3_; scan rate: 0.1 V s^−1^.

The redox behavior of *syn*-tetramer **4b** was explored. The cyclic voltammogram of **4b** was measured in CH_2_Cl_2_ with tetrabutylammonium hexafluorophosphate as the supporting electrolyte (Fig. 4 and S20†). The ferrocene/ferrocenium couple (Fc/Fc^+^) was used as an external reference. In the sweep from −0.60 V to 0.81 V, two peaks were observed at 0.43 and 0.56 V. These values are comparable to the first oxidation peak of porphyrin Ni(ii) complexes (*ca.* 0.58 V).^8^ In the case of porphyrin, the subsequent back-sweep generates a reduction wave at *ca.* 0.38 V. In contrast to porphyrin, *syn*-tetramer **4b** displayed reduction peaks at much lower values (0.10 and −0.29 V). The large displacement between the oxidation and reduction peaks implies the occurrence of dynamic structural changes.^5,6^

#### Oxidative titration

**Fig. 5:**
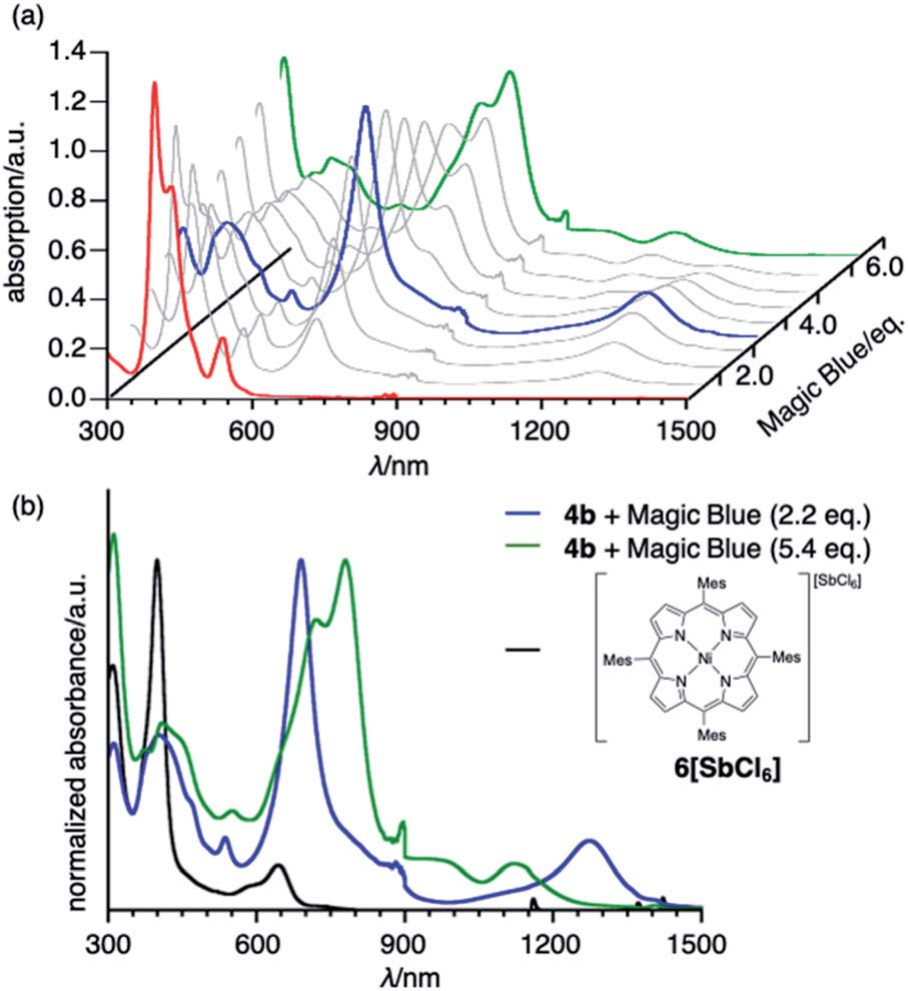
(a) Oxidative titration of **4b** with Magic Blue. [**4b**]_0_ = 3.0 × 10^−6^ M^−1^. (b) UV/vis/NIR absorption spectra of **4b** with 2.2 equiv. of Magic Blue, **4b** with 5.4 equiv. of Magic Blue, and porphyrin radical cation **6[SbCl6]**. Solvent: CH_2_Cl_2_; *λ*: wavelength.

To obtain insight into the unique redox-response of *syn*-tetramer **4b**, we conducted an oxidative titration with tris(4-bromophenyl)aminium hexachloroantimonate (Magic Blue) in CH_2_Cl_2_ while measuring its absorption spectra, which demonstrated two-step spectral changes (Fig. 5). Clear isosbestic points were observed in both cases (Fig. S23†). The first change occurred after the consumption of *ca.* 2 equiv. of Magic Blue, resulting in the appearance of new peaks at 690 and 1274 nm. The further addition of Magic Blue (*ca.* 2 equiv.) resulted in the second change, which led to a slight blue shift of the absorption tail to *ca.* 1250 nm. Similar spectral changes were observed during spectroelectrochemical measurements from 0 to 1.2 V (Fig. S21†). Furthermore, the subsequent back-sweep to −0.5 V recovered the absorption peaks of *syn*-tetramer **4b**.

#### Isolation of the dication

**Scheme 2:**
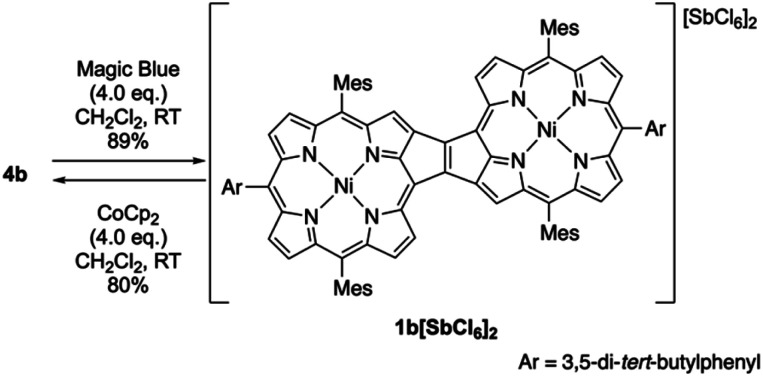
Synthesis of etheno-fused diporphyrin dication **1b[SbCl6]2**.

**Fig. 6:**
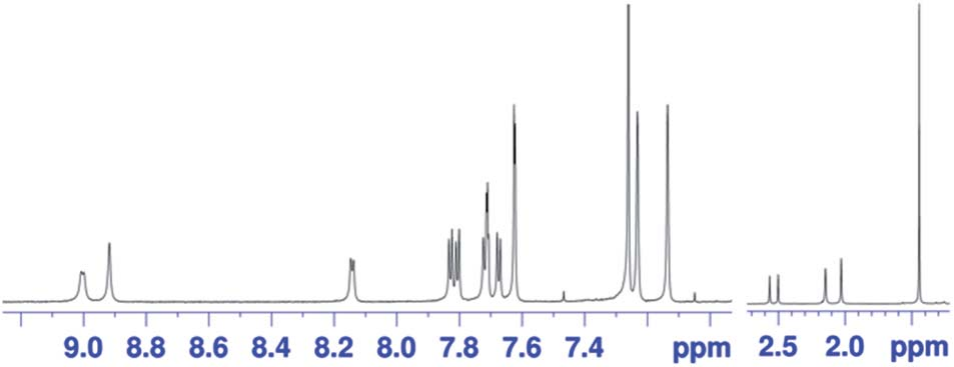
^1^H NMR spectrum of **1b[SbCl6]2** in CDCl_3_ at 25 °C.

**Fig. 7:**
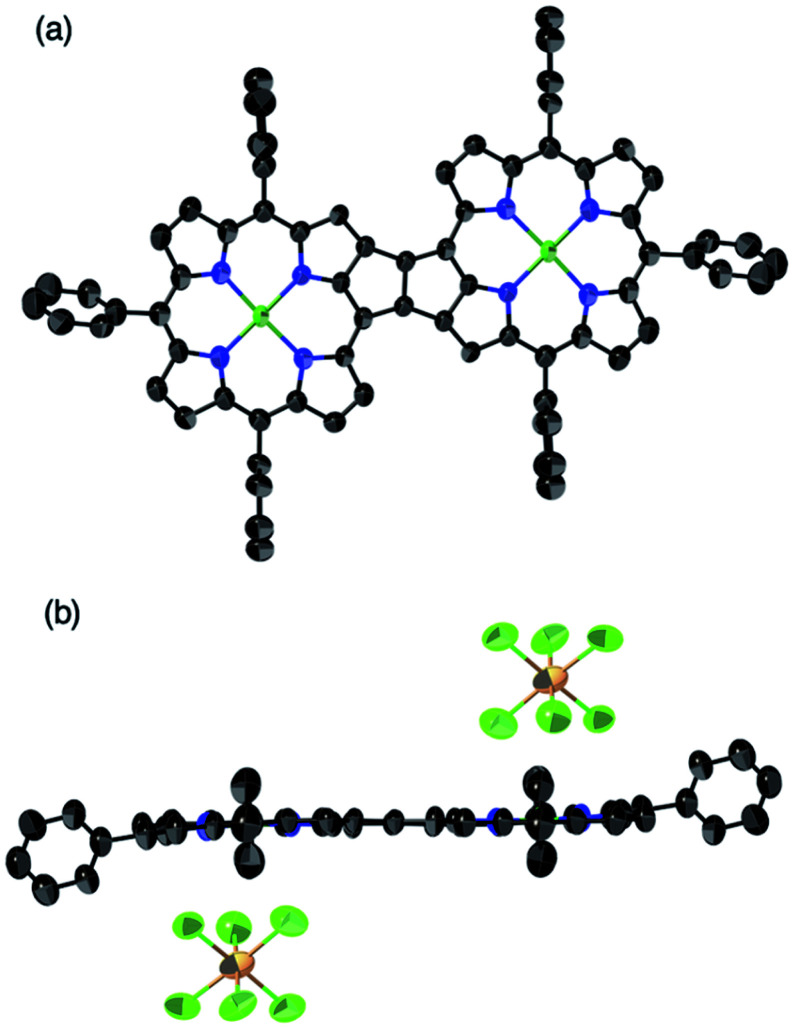
Single-crystal X-ray diffraction structure of etheno-fused diporphyrin dication **1b[SbCl6]2**. (a) Top and (b) side view. Thermal ellipsoids are drawn at 50% probability. Solvent molecules, methyl groups, *tert*-butyl groups, and all hydrogen atoms are omitted for clarity.

The oxidation of *syn*-tetramer **4b** with 4 equiv. of Magic Blue in CH_2_Cl_2_ furnished etheno-fused diporphyrin dication **1b[SbCl6]2** in 89% yield (Scheme 2). The reduction of **1b[SbCl6]2** with an excess of cobaltocene recovered **4b** in 80% yield. While dication **1b[SbCl6]2** is sufficiently stable under ambient conditions, repeated recrystallizations were required for its purification, given that **1b[SbCl6]2** decomposes on silica gel. The ^1^H NMR spectrum of **1b[SbCl6]2** in CDCl_3_ exhibited one singlet and six doublets (7.67–9.00 ppm) due to the main skeleton (Fig. 6). The calculated nucleus-independent chemical shift (NICS)^9^ values of **1b[SbCl6]2** nicely coincide with this observation (Fig. S27†). The presence of a distinct diatropic ring current in **1b[SbCl6]2** can be rationalized by the removal of two electrons from the antiaromatic contribution of the etheno-fused diporphyrin core. Importantly, the signals due to the *ortho*-methyl groups of the mesityl substituents are observed as two singlet signals. Considering that **1b[SbCl6]2** contains two magnetically inequivalent mesityl groups, this result indicates that each set of *ortho*-methyl groups is magnetically equivalent, supporting that **1b[SbCl6]2** adopts a planar structure. The overall structure of **1b[SbCl6]2** was determined based on single-crystal X-ray diffraction analysis, although the crystal data were not of sufficient quality to allow a detailed structural analysis (Fig. 7). Dication **1b[SbCl6]2** adopts a completely planar structure with a mean plane deviation of 0.07 Å. The UV/vis/NIR absorption spectrum of **1b[SbCl6]2** is in good agreement with that observed after the electrochemical oxidation of *syn*-tetramer **4b** (Fig. S21 and S22†).

#### Proposed dyrex-mechanism

The absorption spectrum after the addition of 2.2 equiv. of Magic Blue is clearly different from that of a porphyrin radical cation (Fig. 5b).^10^ Considering that the inter-porphyrin interaction in *syn*-tetramer **4b** is essentially negligible due to the non-conjugative nature of the central cyclobutane unit, the initial change in absorption during the titration cannot be explained by the simple oxidation of **4b**. Furthermore, this absorption is in good agreement with a theoretical simulation of the radical cation of etheno-fused diporphyrin **1b˙** (Fig. S25†).

**Scheme 3:**
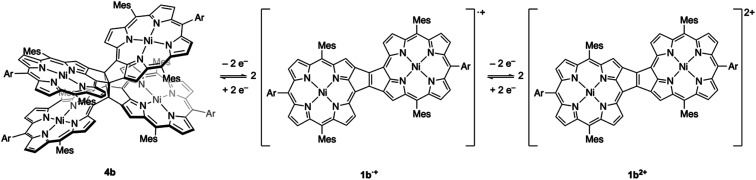
Dyrex response of *syn*-tetramer **4b**.

Based on the results discussed above, we propose the following redox-response process for *syn*-tetramer **4b** (Scheme 3). The first two-electron oxidation of **4b** induces the cleavage of two C–C bonds at the central cyclobutane unit, affording two etheno-fused diporphyrin radical cations **1b˙+**. The subsequent oxidation of **1b˙+** furnishes dication **1b2+**. The reverse reduction process begins with the one-electron reduction of dication **1b2+**. Our previous DFT calculations have predicted that the HOMO level of the etheno-fused diporphyrin is higher than that of a normal porphyrin due to the extended π-system and potential antiaromaticity.^4^ Hence, the reduction potential of **1b2+** can be expected to be much lower than the oxidation potential of **4b**, which would result in a large hysteresis in the cyclic voltammogram. Notably, we have also monitored a similar response during the electrochemical reduction of **4b** (Fig. S20†). However, the identification of the reduced species was unsuccessful owing to the instability of these intermediates.

Importantly, the redox response of **4b**, namely the reversible cycloreversion involving the cleavage of two C–C bonds, is exceptional among dyrex systems.^6,7^ Quadricyclane, anthracene-dimer, and acridizinium dimer undergo double C–C bond cleavage upon electron-transfer, providing norbornadiene, anthracene, and acridizinium, respectively.^11–13^ In these cases, however, the reverse C–C bond formation requires light-irradiation. The unique reactivity of the etheno-fused diporphyrin is attributed to the contribution of antiaromaticity in its macrocyclic conjugation. We believe that the current study offers a general insight that antiaromatic molecules^15^ are a promising candidate for the design of novel redox-active functional organic materials including electrochromic dyes, organic batteries, and organic memory devices.

### Thermal and photo-induced cycloreversion of cyclobutane-linked tetraporphyrin

**Scheme 4:**
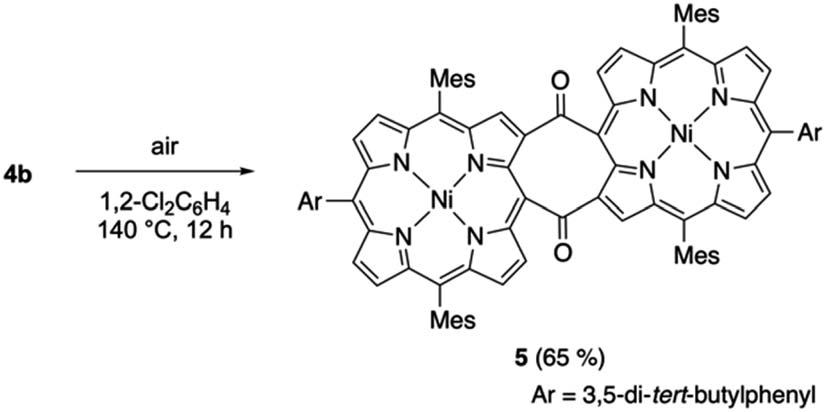
Thermal conversion of *syn*-tetramer **4b** to **5** under aerobic conditions.

We also examined the thermal cycloreversion of *syn*-tetramer **4b**, which was monitored using variable-temperature NMR and UV/vis absorption spectroscopy techniques. The ^1^H NMR spectrum of **4b** in 1,2-dichlorobenzene-*d*_4_ showed slight changes (up to 0.4 ppm) upon increasing the temperature from 20 °C to 120 °C (Fig. S18†). However, the absorption spectrum of **4b** in 1,2-dichlorobenzene displayed negligible changes upon heating (Fig. S19†). Consequently, the temperature-dependent change of the ^1^H NMR chemical shifts can be attributed to the dynamic motion of the *meso*-aryl groups. Notably, heating the dichlorobenzene solution of **4b** to 140 °C afforded diketodiporphyrin **5** in 65% yield (Scheme 4). This result suggests the transient generation of etheno-fused diporphyrin **1b**, which is instantly oxidized to diketone **5**. A similar diketodiporphyrin was formed in our previous study with the corresponding zinc(ii) complexes.^4^ The excited state of nickel(ii) porphyrins generally undergoes a rapid decay through the metal (d,d) state.^14^ Consequently, the formation of diketodiporphyrin **5** implies that the thermally activated triplet state of *in situ*-generated etheno-fused diporphyrin **1b** reacted with triplet oxygen.

We also examined the effect of photo-irradiation on cycloreversion. A CH_2_Cl_2_ solution of **4b** was irradiated by a high-pressure mercury lamp equipped with a sharp cut filter (*λ* > 380 nm) (Fig. S24†). However, no detectable change was observed.

## Conclusions

We have prepared X-shaped cyclobutane-linked tetraporphyrin **4b** and examined the thermal and redox-mediated cycloreversion of its cyclobutane-ring. Heating **4b** in 1,2-dichlorobenzene resulted in negligible changes in the ^1^H NMR and UV/vis absorption spectra. Instead, *syn*-tetramer **4b** undergoes a two-step four-electron oxidation to afford etheno-fused diporphyrin dication **1b2+**. This redox-mediated cyclobutane-ring cycloreversion proceeds in a reversible manner and exhibits a large hysteresis in the cyclic voltammogram. Importantly, this process is accompanied by the cleavage of two C–C bonds, which is exceptional among dyrex systems. The current research highlights the unique reactivity of antiaromatic molecules and offers fundamental insights for the design of novel redox-active functional organic materials including electrochromic dyes, organic batteries, and organic memory devices.

## Author contributions

The manuscript was written through contributions of all authors. All authors have approved the final version of the manuscript. H. S. supervised the project and contributed to conceptualization, project administration, and writing (review & editing) the manuscript. K. M. carried out the synthesis and characterization. I. H. collected the X-ray data of **4b**. N. F. wrote the original draft.

## Conflicts of interest

There are no conflicts to declare.

## Supplementary Material

SC-012-D1SC00438G-s001

SC-012-D1SC00438G-s002
