# Supplementary material for: Redox-induced reversible [2 + 2] cycloaddition of an etheno-fused diporphyrin
Source: Chem Sci. 2021 Feb 24;12(14):5224–9. doi: 10.1039/d1sc00438g (PMC8179634; doi:10.1039/d1sc00438g)
Supplement: SC-012-D1SC00438G-s001 [file SC-012-D1SC00438G-s001.pdf]

Electronic Supplementary Material (ESI) for Chemical Science.
This journal is © The Royal Society of Chemistry 2021

Electronic Supporting Information

Redox-Induced Reversible [2+2] Cycloaddition of an Etheno-Fused Diporphyrin

Kazuya Miyagawa,[1] Ichiro Hisaki,[2] Norihito Fukui,[1] and Hiroshi Shinokubo*[1]

[1]Department of Molecular and Macromolecular Chemistry, Graduate School of Engineering, Nagoya University, Furo-cho, Chikusa-ku, Nagoya 464-8603 (Japan)

[2]Graduate School of Engineering Science, Osaka University, 1-3 Machikaneyama, Toyonaka, Osaka 560-8531 (Japan)

E-mail: hshino@chembio.nagoya-u.ac.jp

Table of Contents

1. Instrumentation and materials 3

2. Experimental procedures and compound data 4

3. NMR spectra 13

4. Crystal data 29

5. Temperature-dependent ^{1}H NMR spectra 31

6. Temperature-dependent UV-vis absorption spectra 32

7. Cyclic voltammograms 33

8. Spectroelectrochemistry 34

9. UV/vis/NIR absorption spectra 35

10. Photostability 36

11. DFT calculations 37

12. References 41

1. Instrumentation and materials

^{1}H NMR (500 MHz) and ^{13}C NMR (126 MHz) spectra were recorded on a Varian INOVA-500 spectrometer. Chemical shifts were reported as the delta scale in ppm relative to $CHCl_3$ (δ = 7.26 ppm) for ^{1}H NMR and $CDCl_3$ (δ = 77.16 ppm) for ^{13}C NMR.

UV/vis/NIR absorption spectra were recorded on a Shimadzu UV-2550 or JASCP V 670 spectrometer.

High-resolution atmospheric pressure chemical ionization time-of-flight (APCI-TOF) and electrospray ionization time-of-flight (ESI-TOF) mass spectra were taken on a Bruker micrOTOF instrument using a positive ionization mode. High-resolution matrix assisted laser desorption and ionization time-of-flight (MALDI-TOF) mass spectra were taken on a Bruker autoflex max using a positive ionization mode.

X-ray data were obtained using a Rigaku CCD diffractometer (Saturn 724 with MicroMax-007) with Varimax Mo optics.

Cyclic voltammograms were obtained under the following conditions; solvent: CH_2Cl_2, electrolyte: 0.1 M Bu_4NPF_6, working electrode: glassy carbon, counter electrode: Pt, reference electrode: $Ag/AgNO_3$, scan rate: 0.1 V/s.

Photo-irradiation was conducted by the USHIO OPM2-252H lamp house equipped with a super-high pressure mercury lamp (250 W) and a sharp cut filter L38.

All calculations were carried out using the *Gaussian 09* software package.[1] Initial geometry for the calculations of **1b**$^{+}$ and **1b**$^{2+}$ were obtained from the X-ray crystal structure of **1b[SbCl$_6$]$_2$**. Aryl groups at the *meso*-positions were omitted to simplify the calculations. The structures were optimized by density functional theory (DFT) method with B3LYP level, employing basis sets 6-31G(d) for C, H, and N and SDD for Ni. The oscillator strengths of **1b**$^{+}$ and **1b**$^{2+}$ were computed by the TD-DFT formalism at the B3LYP/6-31G(d)+SDD level.

Tetrahydrofuran (THF) was purchased from Wako Pure Chemical Industries, Ltd. as a dehydrated grade. Dry CH_2Cl_2 was prepared by distillation from CaH_2. Unless otherwise noted, materials obtained from commercial suppliers were used without further purification.

2. Experimental procedures and compound data

2-Iodo-10-(3,5-di-*tert*-butylphenyl)-5,15-dimesitylporphyrinato nickel(II) S2

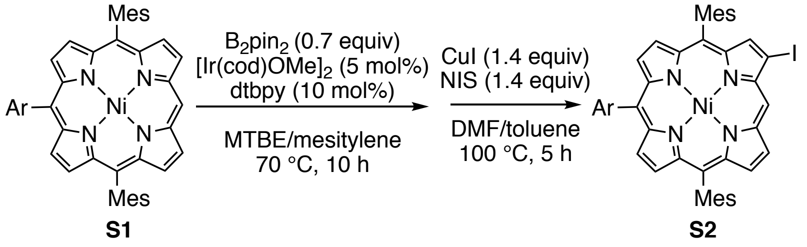

A Schlenk tube containing **S1**[2] (1.0 g, 1.3 mmol), B_2pin_2 (0.23 g, 0.88 mmol), $[Ir(cod)OMe]_2$ (42 mg, 0.063 mol), and 4,4'-di-*tert*-butyl-2,2'-bipyridyl (dtbpy, 34 mg, 0.13 mmol) was filled with N_2, and then charged with mesitylene (13 mL) and methyl *tert*-butyl ether (MTBE, 38 mL). After stirring at 70 °C for 10 h, the reaction mixture was passed through short silica gel column eluting with CH_2Cl_2. After removal of the solvent *in vacuo*, the crude mixture was dissolved in *N*,*N*-dimethylformamide (DMF, 80 mL) and toluene (40 mL). To the resulting solution, *N*-iodosuccinimide (NIS, 0.40 g, 1.8 mmol) and CuI (0.34 g, 1.8 mmol) were added. After stirring at 100 °C for 5 h, the reaction mixture was quenched with aqueous NH_4Cl solution, extracted with toluene, washed with aqueous $Na_2S_2O_3$ solution, water, and brine, and dried over Na_2SO_4. After removal of the solvent *in vacuo*, the residue was separated by silica gel chromatography eluting with CH_2Cl_2/hexane (1:20). After removal of the solvent *in vacuo*, recrystallization from CH_2Cl_2/MeOH gave **S2** (0.97 g, 1.1 mmol, 84%).

^{1}H NMR (500 MHz, $CDCl_3$, 298 K): δ = 9.86 (s, 1H, *meso*), 9.16 (d, J = 5.0 Hz, 1H, β), 8.94 (s, 1H, β), 8.77 (d, J = 5.0 Hz, 1H, β), 8.76 (d, J = 5.0 Hz, 1H, β), 8.72 (d, J = 5.0 Hz, 1H, β), 8.63 (d, J = 5.0 Hz, 1H, β), 8.61 (d, J = 5.0 Hz, 1H, β), 7.90 (d, J = 1.5 Hz, 2H, Ar-*o*), 7.25 (t, J = 2.0 Hz, 1H, Ar-*p*), 7.23 (s, 4H, Mes), 2.59 (s, 3H, Mes), 2.59 (s, 3H, Mes), 1.81 (s, 6H, Mes), 1.81 (s, 6H, Mes), 1.47 (s, 18H, *t*-Bu) ppm; ^{13}C NMR (126 MHz, $CDCl_3$, 298 K): δ = 149.0, 144.0, 143.5, 143.5, 143.2, 143.1, 142.7, 142.5, 141.5, 140.1, 139.3, 139.2, 139.1, 138.0, 137.9, 137.2, 137.0, 133.2, 133.2, 133.1, 131.8, 131.4, 131.3, 129.1, 128.0, 127.9, 121.2, 120.9, 117.5, 116.4, 105.0, 91.1, 35.2, 31.8, 21.5, 21.5 ppm (Some signals due to alkyl groups were overlapped.); HRMS (APCI): $[M+H]^+$ Calcd for $C_{52}H_{51}IN_4Ni$ 917.2585; Found 917.2544.

2-Iodo-10-(3,5-di-*tert*-butylphenyl)-5,15-dimesitylporphyrin S3

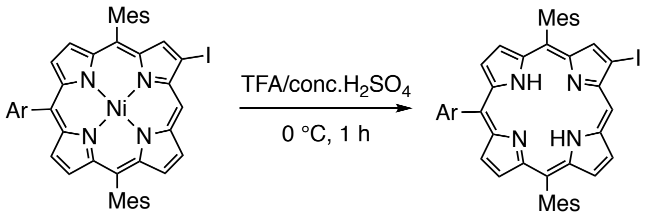

S2 **S3**

Conc. H_2SO_4 (13 mL) was added slowly to a mixture of **S2** (3.3 g, 3.6 mmol) and trifluoroacetic acid (TFA, 50 mL) at 0 °C. After stirring at 0 °C for 1 h, the reaction mixture was quenched with an equimolar amount of NaOH solution at 0 °C. The product was extracted with CH_2Cl_2, washed with brine, and dried over Na_2SO_4. After removal of the solvent *in vacuo*, the residue was separated by silica gel chromatography eluting with CH_2Cl_2/hexane (1:4). After removal of the solvent *in vacuo*, recrystallization from CH_2Cl_2/MeOH gave **S3** (2.2 g, 2.6 mmol, 73%).

^{1}H NMR (500 MHz, $CDCl_3$, 298 K): δ = 10.19 (s, 1H, *meso*), 9.40 (d, J = 4.5 Hz, 1H, β), 8.97 (s, 1H, β), 8.92 (d, J = 5.0 Hz, 1H, β), 8.88 (d, J = 4.5 Hz, 1H, β), 8.77 (d, J = 4.5 Hz, 1H, β), 8.77 (d, J = 4.5 Hz, 1H, β), 8.64 (d, J = 4.5 Hz, 1H, β), 8.06 (d, J = 2.0 Hz, 2H, Ar-*o*), 7.79 (t, J = 1.8 Hz 1H, Ar-*p*), 7.30 (s, 4H, Mes), 2.64 (s, 3H, Mes), 2.64 (s, 3H, Mes), 1.85 (s, 6H, Mes), 1.84 (s, 6H, Mes), 1.51 (s, 18H, *t*-Bu), –2.83 (s, 2H, NH) ppm; ^{13}C NMR (126 MHz, $CDCl_3$, 298 K): δ = 148.7, 148.6, 141.0, 140.4, 139.5, 139.4, 139.4, 138.2, 138.0, 137.9, 137.9, 137.7, 137.7, 135.1, 132.6, 129.9, 129.6, 129.2, 127.9, 127.8, 127.8, 127.4, 121.8, 121.4, 121.1, 121.0, 118.2, 117.6, 117.3, 103.8, 35.1, 31.7, 21.7, 21.7, 21.5 ppm (Some signals due to alkyl groups were overlapped. Some signals due to aromatic protons are too broad to be observed because of the amine-imine tautomerization.); HRMS (APCI): $[M+H]^+$ Calcd for $C_{52}H_{53}IN_4$ 861.3388; Found 861.3346.

1,2-Bis(2-(10-bis(3,5-di-*tert*-butylphenyl)-5,15-mesityl)porphyrinyl)acetylene S4

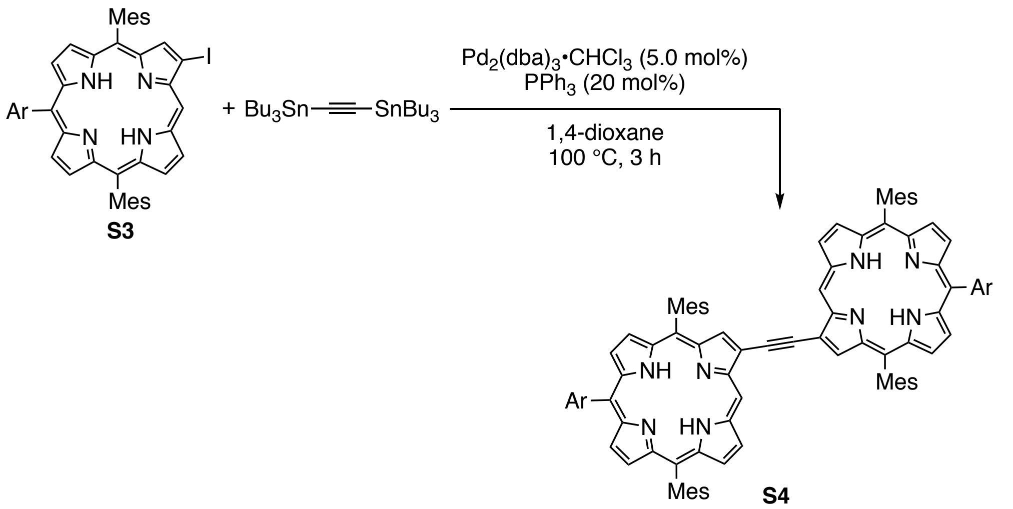

A two-necked, stoppered round bottomed flask containing **S3** (0.50 g, 0.58 mmol), $Pd_2(dba)_3$•$CHCl_3$ (31 mg, 29 μmol), and PPh_3 (30 mg, 0.12 mmol) was filled with N_2, and then charged with dry 1,4-dioxane (30 mL). To the solution, bis(tributylstannyl)ethyne (0.15 mL, 0.18 g, 0.290 mmol) was added. After stirring at 100 °C for 12 h, the reaction mixture was passed through short silica column eluting with toluene. After removal of the solvent *in vacuo*, the residue was purified by silica gel chromatography eluting with CH_2Cl_2/hexane (1/3). After removal of the solvent *in vacuo*, recrystallization from CH_2Cl_2/methanol gave **S4** (0.29 g, 0.19 mmol, 67%).

^{1}H NMR (500 MHz, toluene-d_8/CS_2 (1/1), 298 K): δ = 10.79 (s, 2H, *meso*), 9.23 (s, 2H, β), 9.12 (d, J = 4.5 Hz, 2H, β), 8.81 (d, J = 5.0 Hz, 2H, β), 8.70 (d, J = 4.5 Hz, 2H, β), 8.69 (d, J = 4.5 Hz, 2H, β), 8.67 (d, J = 4.5 Hz, 2H, β), 8.54 (d, J = 4.5 Hz, 2H, β), 7.99 (d, J = 1.5 Hz, 4H, Ar-*o*) 7.72 (t, J = 1.8 Hz, 2H, Ar-*p*), 7.20 (s, 4H, Mes), 7.13 (s, 4H, Mes), 2.54 (s, 6H, Mes), 2.49 (s, 6H, Mes), 1.91 (s, 12H, Mes), 1.82 (s, 12H, Mes), 1.43 (s, 36H, *t*-Bu), –2.44 (s, 4H, NH) ppm; ^{13}C NMR (126 MHz, toluene-d_8/CS_2 (1/1), 298 K): δ = 148.9, 142.1, 139.8, 139.6, 138.7, 138.5, 138.2, 137.9, 137.7, 137.3, 137.2, 135.3, 132.7, 130.2, 129.3, 128.7, 128.5, 128.4, 127.7, 125.6, 124.8, 122.0, 121.4, 118.7, 118.2, 103.1, 95.2, 35.2, 32.1, 22.2, 22.2, 22.1, 22.0 ppm (Some signals due to aromatic protons are too broad to be observed because of the amine-imine tautomerization.); HRMS (ESI): $[M]^+$ Calcd for $C_{106}H_{106}N_8$ 1492.8646; Found 1492.8700.

1,2-Bis(2-(10-bis(3,5-di-*tert*-butylphenyl)-5,15-mesityl)Zn(II)porphyrinyl)acetylene S5

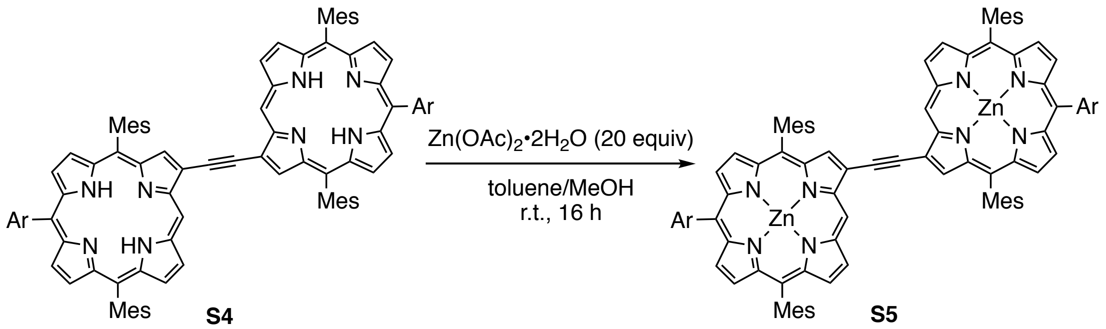

S4 S5

To a solution of **S4** (0.17 g, 0.11 mmol) in toluene (30 mL) was added a solution of zinc acetate dihydrate (0.49 g, 2.3 mmol) in MeOH (6.0 mL). After stirring at room temperature for 16 h, the reaction mixture was passed through short silica column eluting with toluene. After removal of the solvent *in vacuo*, the residue was purified by silica gel chromatography eluting with CH_2Cl_2/hexane (2/5). After removal of the solvent *in vacuo*, recrystallization from $CHCl_3$/CH_3CN gave **S5** (0.15 g, 0.93 mmol, 83%).

^{1}H NMR (500 MHz, toluene-d_8/CS_2 (1/1), 298 K): δ = 10.98 (s, 2H, *meso*), 9.48 (s, 2H, β), 9.33 (d, J = 4.5 Hz, 2H, β), 8.95–8.97 (m, 4H, β), 8.90 (d, J = 4.5 Hz, 2H, β), 8.84 (d, J = 4.5 Hz, 2H, β), 8.79 (d, J = 4.5 Hz, 2H, β), 8.15 (d, J = 2.0 Hz, 4H, Ar-*o*) 7.18 (t, J = 2.0 Hz, 2H, Ar-*p*), 7.31 (s, 4H, Mes), 7.24 (s, 4H, Mes), 2.65 (s, 6H, Mes), 2.60 (s, 6H, Mes), 2.04 (s, 12H, Mes), 1.96 (s, 12H, Mes), 1.54 (s, 36H, *t*-Bu) ppm; ^{13}C NMR (126 MHz, toluene-d_8/CS_2 (1/1), 298 K): δ = 151.1, 150.9, 150.8, 150.8, 150.4, 150.3, 148.8, 148.8, 142.7, 139.6, 139.5, 139.4, 137.9, 137.7, 137.3, 137.2, 133.6, 133.4, 131.8, 131.2, 131.1, 130.2, 128.5, 127.7, 127.3, 125.7, 124.8, 122.7, 121.2, 119.7, 119.1, 104.3, 94.9, 35.2, 32.1, 22.4, 22.4, 22.1, 22.0 ppm; HRMS (MALDI): $[M]^+$ Calcd for $C_{106}H_{102}N_8Zn_2$ 1618.6798; Found 1618.6845.

1,2-Bis(2-(20-bromo-10-bis(3,5-di-*tert*-butylphenyl)-5,15-mesityl)Ni(II)porphyrinyl)acetylene 2b

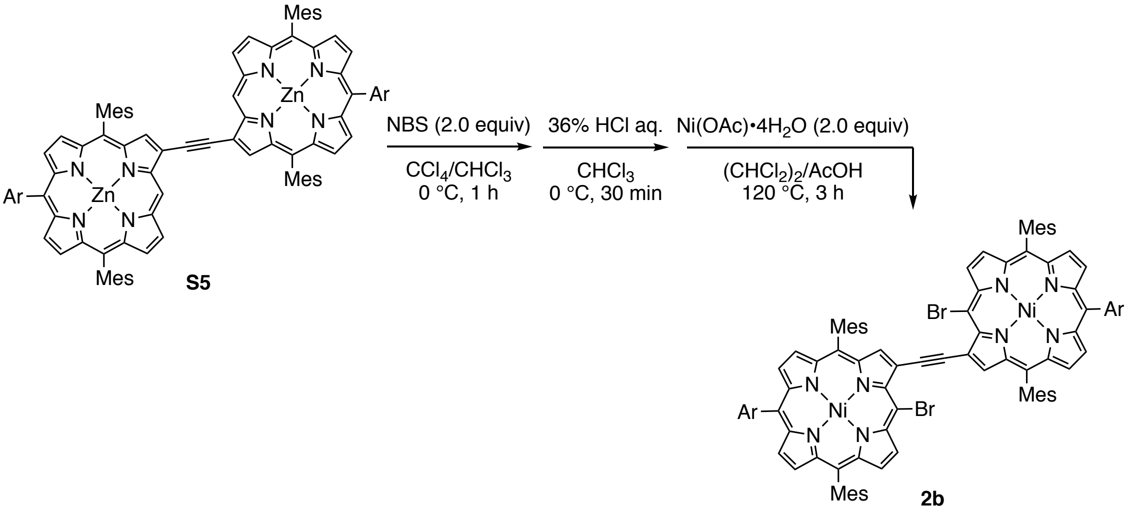

A round-bottomed flask containing **S5** (0.29 g, 0.180 mmol) was charged with CCl_4 (150 mL) and $CHCl_3$ (150 mL). The reaction mixture was cooled to 0 °C. To the flask, a solution of *N*-bromosuccinimide (64 mg, 0.360 mmol) in $CHCl_3$ (30 mL) was added. After stirring at 0 °C for 1 h, the reaction mixture was quenched with a sufficient amount of acetone. The product was passed through alumina pad eluting with $CHCl_3$. After removal of the solvent *in vacuo*, the crude mixture was dissolved in $CHCl_3$ (270 mL). To the resulting solution, 36% HCl aq. (18 drops) was added. After stirring at room temperature for 30 min, the reaction mixture was quenched with a sufficient amount of $NaHCO_3$ solution at 0 °C. The product was extracted with $CHCl_3$, washed with brine, and dried over Na_2SO_4. After removal of the solvent *in vacuo*, the residue passed through short silica column eluting with $CHCl_3$. After removal of the solvent *in vacuo*, the crude mixture was dissolved in 1,1,2,2-tetrachloroethane (100 mL) and AcOH (10 mL). To the resulting solution, nickel(II) acetate tetrahydrate (0.50 g, 2.0 mmol) was added. After stirring at 120 °C for 3 h, the reaction mixture was quenched with a sufficient amount of $NaHCO_3$ solution. The product was extracted with $CHCl_3$, washed with brine, and dried over Na_2SO_4. After removal of the solvent *in vacuo*, the residue was purified by silica gel chromatography eluting with CH_2Cl_2/hexane (1/5). After removal of the solvent *in vacuo*, recrystallization from CH_2Cl_2/MeOH gave **2b** (0.23 g, 0.13 mmol, 74%).

^{1}H NMR (500 MHz, $CDCl_3$, 298 K): δ = 9.65 (d, J = 4.5 Hz, 2H, β), 9.13 (s, 2H, β), 8.75–8.71 (m, 4H, β), 8.63 (d, J = 5.0 Hz, 2H, β), 8.57 (d, J = 4.5 Hz, 2H, β), 8.55 (d, J = 4.5 Hz, 2H,

β), 7.91 (d, *J* = 2.0 Hz, 4H, Ar-*o*) 7.75 (t, *J* = 1.5 Hz, 2H, Ar-*p*), 7.28 (s, 4H, Mes), 7.24 (s, 4H, Mes), 2.61 (s, 6H, Mes), 2.59 (s, 6H, Mes), 1.96 (s, 12H, Mes), 1.90 (s, 12H, Mes), 1.51 (s, 36H, *t*-Bu) ppm; ^{13}C NMR (126 MHz, $CDCl_3$, 298 K): δ = 149.2, 144.0, 143.8, 143.6, 142.8, 142.8, 142.6, 140.4, 139.6, 139.6, 139.1, 139.1, 138.1, 138.0, 137.7, 136.7, 136.7, 135.1, 133.7, 133.5, 132.4, 132.1, 131.9, 128.9, 128.1, 128.0, 128.0, 121.4, 120.5, 118.0, 117.8, 101.6, 97.9, 35.2, 31.8, 21.6, 21.6, 21.5, 21.5 ppm; HRMS (MALDI): $[M]^+$ Calcd for $C_{106}H_{100}{}^{79}Br_2N_8Ni_2$ 1762.5122; Found 1762.5147.

Cyclobutane-linked tetraporphyrin 4b

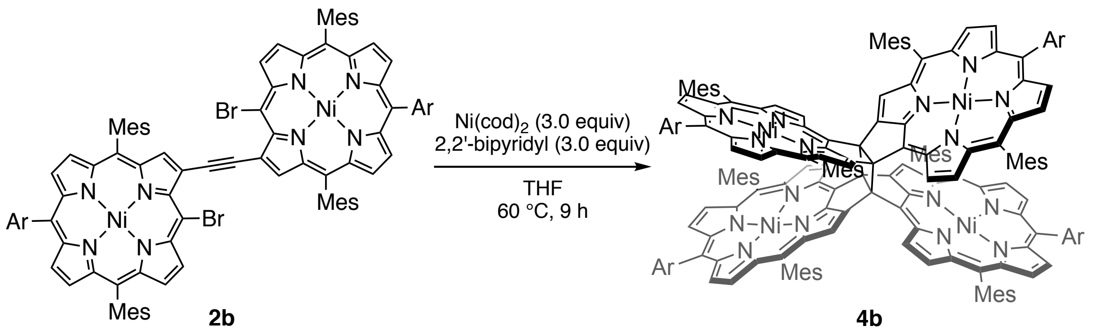

A Schlenk tube containing **2b** (53 mg, 30 μmol) was charged with dry THF (9.0 mL). To the tube, a solution of bis(1,5-cyclooctadiene)nickel(0) (26 mg, 90 μmol) and 2,2'-bipyridyl (14 mg, 90 μmol) in dry THF (9.0 mL) was added. The reaction mixture was stirred at 60 °C for 9 h. After removal of the solvent *in vacuo*, the residue was separated by silica gel chromatography eluting with CH_2Cl_2/hexane (1:5). After removal of the solvent *in vacuo*, recrystallization from CH_2Cl_2/MeOH gave **4b** (9.1 mg, 2.8 μmol, 57%).

^{1}H NMR (500 MHz, $CDCl_3$, 298 K): δ = δ = 10.17 (d, *J* = 4.5 Hz, 4H, *β*), 8.95 (s, 4H, *β*), 8.49 (d, *J* = 4.5 Hz, 4H, *β*), 8.38 (d, *J* = 4.5 Hz, 4H, *β*), 8.36 (d, *J* = 4.5 Hz, 4H, *β*), 8.20–8.22 (m, 8H, *β*), 7.83 (s, 4H, Ar-*o*), 7.56 (s, 4H, Ar-*p*), 7.30 (s, 4H, Ar-*o*), 7.22 (s, 4H, Mes), 7.16 (s, 4H, Mes), 7.09 (s, 4H, Mes), 7.05 (s, 4H, Mes), 2.68 (s, 12H, Mes), 2.62 (s, 12H, Mes), 1.36 (s, 36H, *t*-Bu), 1.31 (s, 12H, Mes), 1.28 (s, 36H, *t*-Bu), 1.24 (s, 12H, Mes), 1.15 (s, 12H, Mes), 1.12 (s, 12H, Mes) ppm; ^{13}C NMR (126 MHz, $CDCl_3$, 298 K): δ = 153.7, 151.0, 148.7, 148.6, 146.0, 143.5, 143.0, 142.7, 142.5, 142.3, 142.3, 140.4, 139.2, 138.9, 138.0, 137.5, 137.4, 136.7, 132.0, 131.8, 131.6, 130.3, 130.1, 129.2, 128.8, 128.2, 127.9, 127.6, 127.6, 121.6, 120.7, 120.2, 118.3, 117.4, 117.3, 76.5, 35.0, 35.0, 31.7, 31.7, 21.7, 21.6, 21.5, 21.4, 21.2, 21.1 ppm (Some signals

were overlapped.); UV/Vis absorption (CH_2Cl_2): λ_{max} (ε/M^{-1} cm^{-1}) = 397 (4.2 × 10^5), 431 (2.8 × 10^5), and 539 (8.0 × 10^4) nm； HRMS (ESI): $[M+H]^+$ Calcd for $C_{212}H_{200}N_{16}Ni_4$ 3206.3650; Found 3206.3571.

Diketodiporphyrin 5

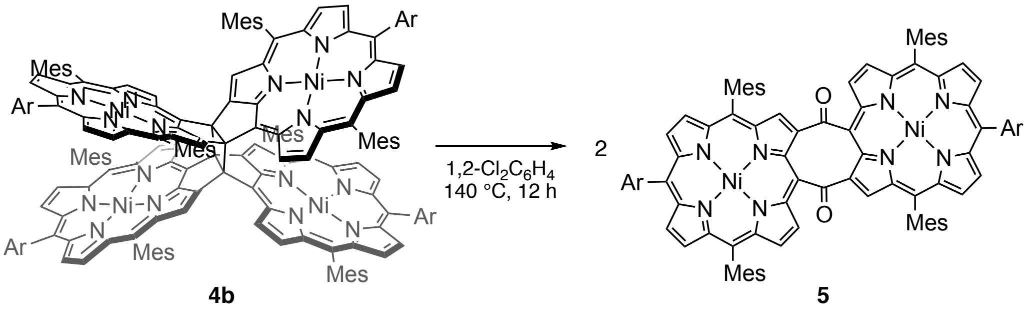

A test tube containing **4b** (5.0 mg, 1.6 μmol) was charged with 1,2-$Cl_2C_6H_4$ (3.0 mL). The tube was heated at 140 °C for 12 h. After cooling to room temperature, hexane (15 mL) was added to the solution. The resulting solution was passed through short silica column eluting with CH_2Cl_2. After removal of the solvent *in vacuo*, the residue was separated by silica gel chromatography eluting with CH_2Cl_2/hexane (2:1). After removal of the solvent *in vacuo*, recrystallization from CH_2Cl_2/MeOH gave **5** (3.3 mg, 2.0 μmol, 65%).

^{1}H NMR (500 MHz, $CDCl_3$, 253 K): δ = 9.61 (d, *J* = 5.0 Hz, 2H, β), 8.73 (s, 2H, β), 8.73 (d, *J* = 5.5 Hz, 2H, β), 8.67 (d, *J* = 5.0 Hz, 2H, β), 8.55 (d, *J* = 5.0 Hz, 2H, β), 8.49 (d, *J* = 5.0 Hz, 2H, β), 8.35 (d, *J* = 5.0 Hz, 2H, β), 8.13 (s, 2H, Ar-*o*), 7.61 (t, *J* = 1.8 Hz, 2H, Ar-*p*), 7.32 (s, 2H, Mes), 7.30 (s, 2H, Ar-*o*), 7.13 (s, 2H, Mes), 7.10 (s, 2H, Mes), 7.06 (s, 2H, Mes), 2.58 (s, 6H, Mes), 2.50 (s, 6H, Mes), 2.20 (s, 6H, Mes), 1.80 (s, 6H, Mes), 1.46 (s, 18H, *t*-Bu), 1.44 (s, 6H, Mes), 1.34 (s, 6H, Mes), 1.26 (s, 18H, *t*-Bu) ppm; ^{13}C NMR (126 MHz, $CDCl_3$, 298 K): δ = 200.2, 192.7, 149.1, 146.8, 144.0, 143.7, 143.6, 143.3, 143.0, 139.8, 139.2, 139.2, 139.1, 138.9, 138.7, 138.2, 138.1, 136.6, 136.0, 134.1, 133.9, 133.5, 133.3, 131.9, 131.7, 131.6, 130.9, 128.8, 128.1, 128.1, 128.0, 127.9, 122.2, 121.4, 119.7, 118.4, 115.3, 35.1, 31.7, 21.7, 21.6, 21.6, 21.5, 21.4, 21.3 ppm (Some signals were overlapped.); HRMS (MALDI): $[M]^+$ Calcd for $C_{106}H_{100}N_8Ni_2O_2$ 1635.6756; Found 1635.67940.

Etheno-fused diporphyrin dication 1b^{2+}

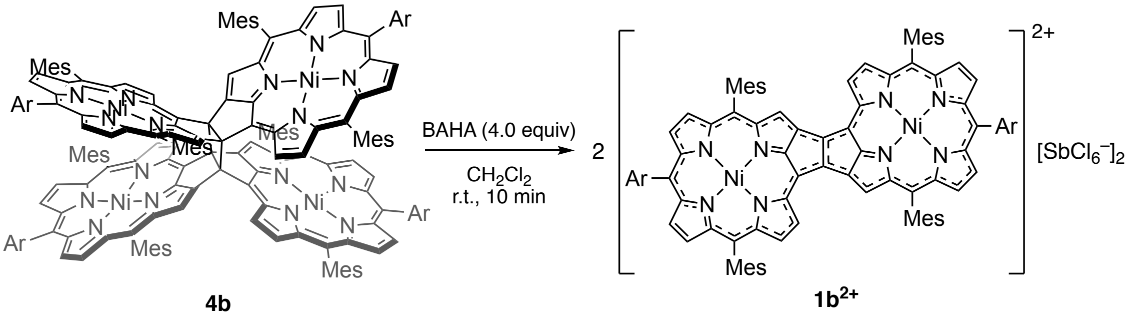

A Schlenk tube containing **4b** (9.6 mg, 3.0 µmol) was charged with CH_2Cl_2 (10 mL). To the solution, tris(4-bromophenyl)aminium hexachloroantimonate (9.8 mg, 12 µmol) was added After stirring at room temperature for 10 min, hexane (10 mL) was added to the solution. After removal of the half amount of solvent *in vacuo*, reprecipitation gave **1b^{2+}** (12 mg, 5.4 µmol, 89%).

^{1}H NMR (500 MHz, $CDCl_3$, 298 K): δ = 9.00 (d, J = 4.0 Hz, 2H, β), 8.92 (s, 2H, β), 8.14 (d, J = 4.5 Hz, 2H, β), 7.83 (d, J = 5.0 Hz, 2H, β), 7.81 (d, J = 5.0 Hz, 2H, β), 7.71–7.72 (m, 4H, β + Ar-*p*), 7.67 (d, J = 5.0 Hz, 2H, β), 7.62 (d, J = 1.5 Hz, 4H, Ar-*o*), 7.23 (s, 4H, Mes), 7.14 (s, 4H, Mes), 2.56 (s, 6H, Mes), 2.50 (s, 6H, Mes), 2.15 (s, 12H, Mes), 2.03 (s, 12H, Mes), 1.44 (s, 36H, *t*-Bu) ppm; ^{13}C NMR (126 MHz, $CDCl_3$, 298 K): δ = 170.5, 167.1, 154.0, 152.9, 151.3, 150.8, 150.5, 150.4, 149.7, 149.1, 140.0, 139.5, 139.4, 138.3, 138.0, 138.0, 137.7, 136.4, 136.1, 134.7, 134.4, 134.2, 133.7, 132.2, 131.8, 131.6, 128.9, 128.5, 127.6, 127.5, 123.3, 109.0, 35.2, 31.7, 21.6, 21.5, 21.5, 21.4 ppm; HRMS (MALDI): $[M]^+$ Calcd for $C_{106}H_{100}N_8Ni_2$ 1602.6779; Found 1602.6708.

Reduction of etheno-fused diporphyrin dication 1b^{2+}

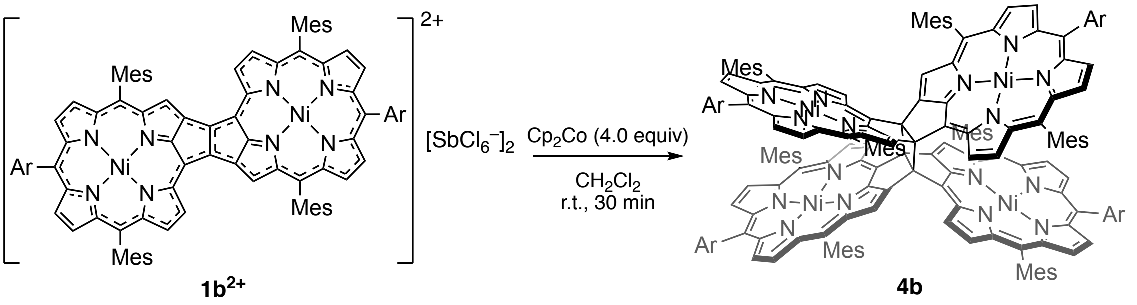

A Schlenk tube containing **1b^{2+}** (4.5 mg, 2.0 µmol) was charged with CH_2Cl_2 (5 mL). To the

solution, bis(cyclopentadienyl)cobalt(II) (1.5 mg, 8.0 μmol) was added. The reaction mixture was stirred at room temperature for 30 min. After removal of the solvent *in vacuo*, the residue was separated by silica gel chromatography eluting with CH_2Cl_2/hexane (1:5). After removal of the solvent *in vacuo*, recrystallization from CH_2Cl_2/MeOH gave **4b** (2.6 mg, 0.80 μmol, 80%).

3. NMR spectra

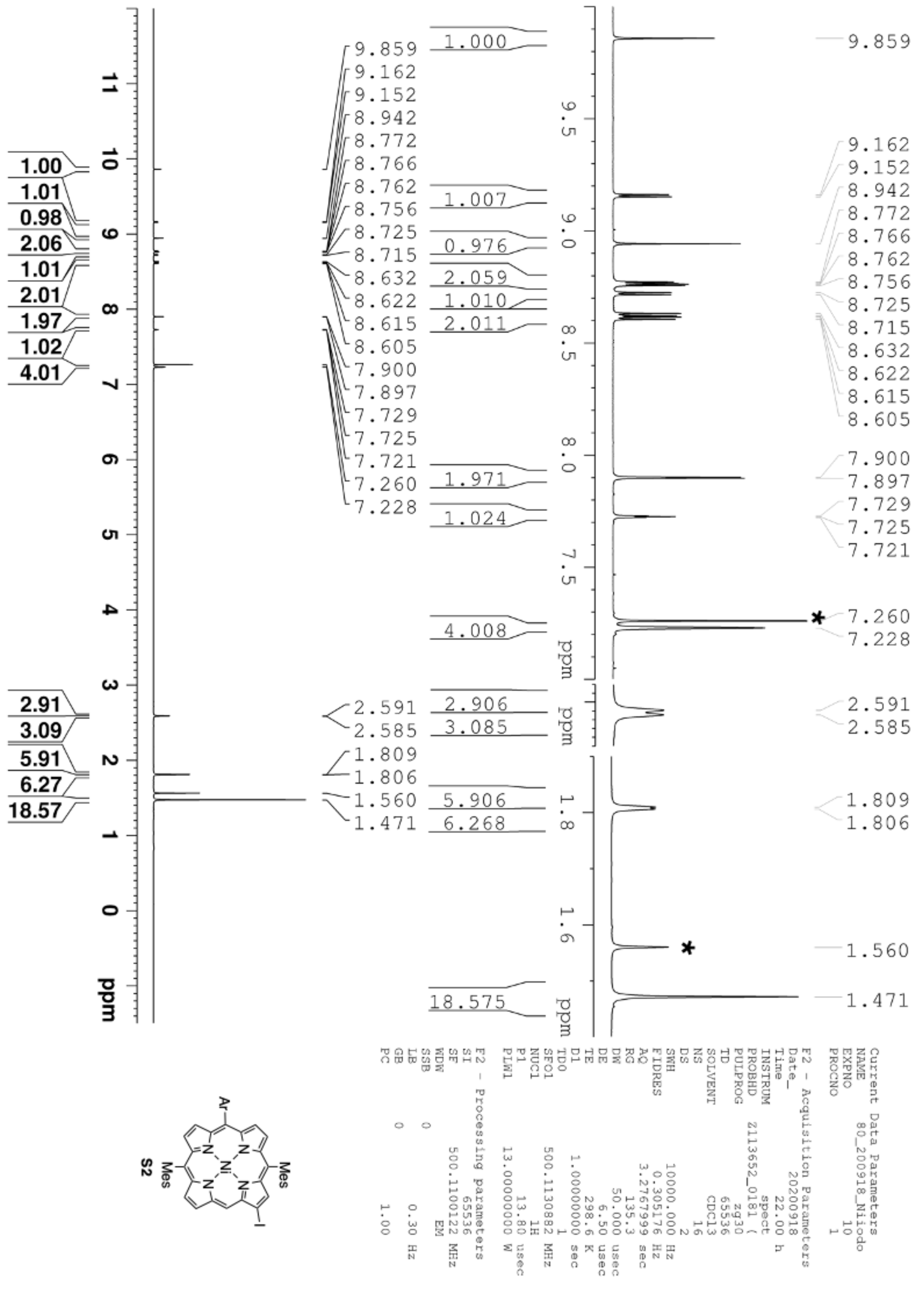

Fig. S1 ^{1}H NMR spectrum of **S2** in $CDCl_3$ at 25 °C.

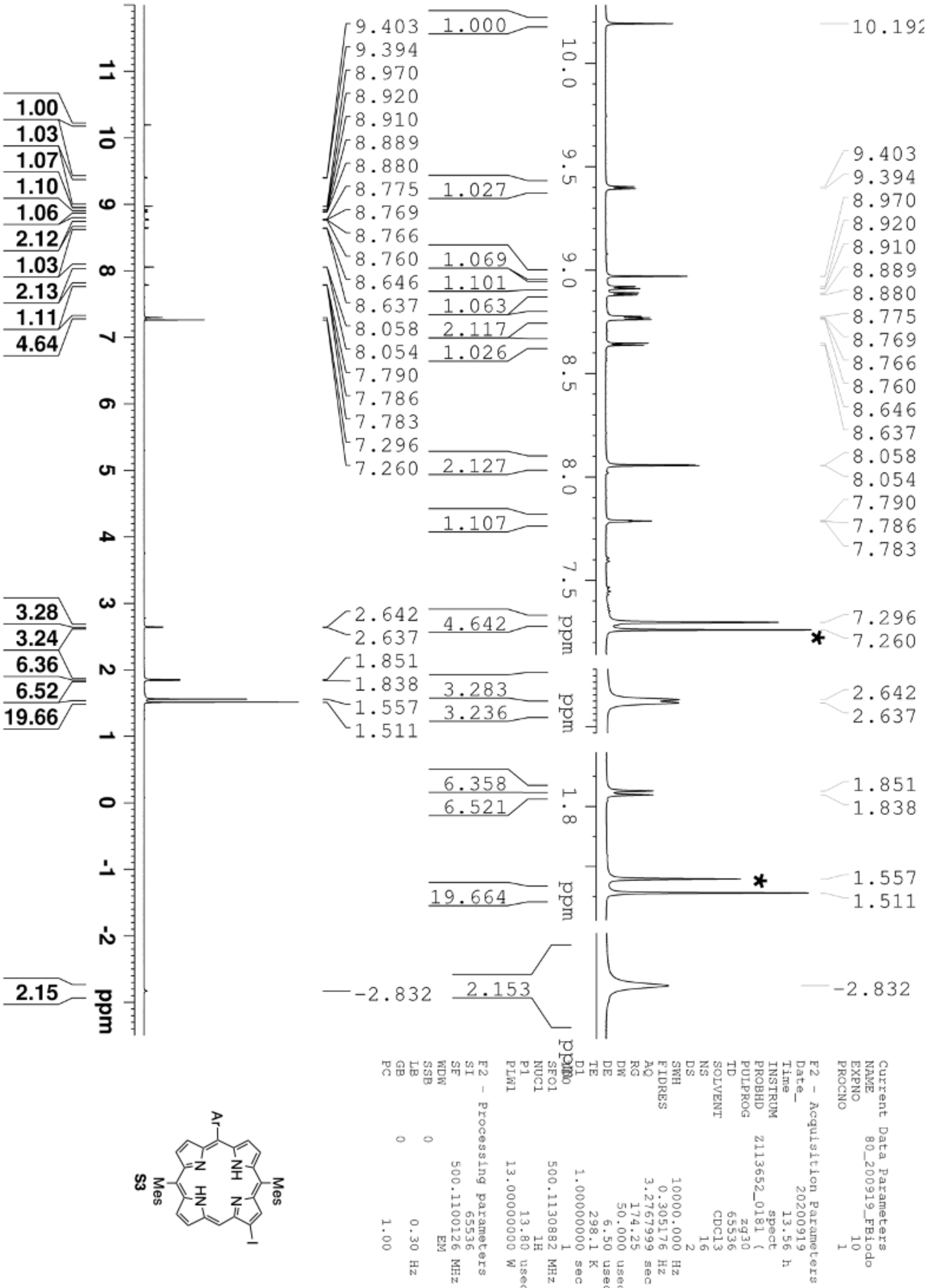

Fig. S2 ^{1}H NMR spectrum of **S3** in $CDCl_3$ at 25 °C.

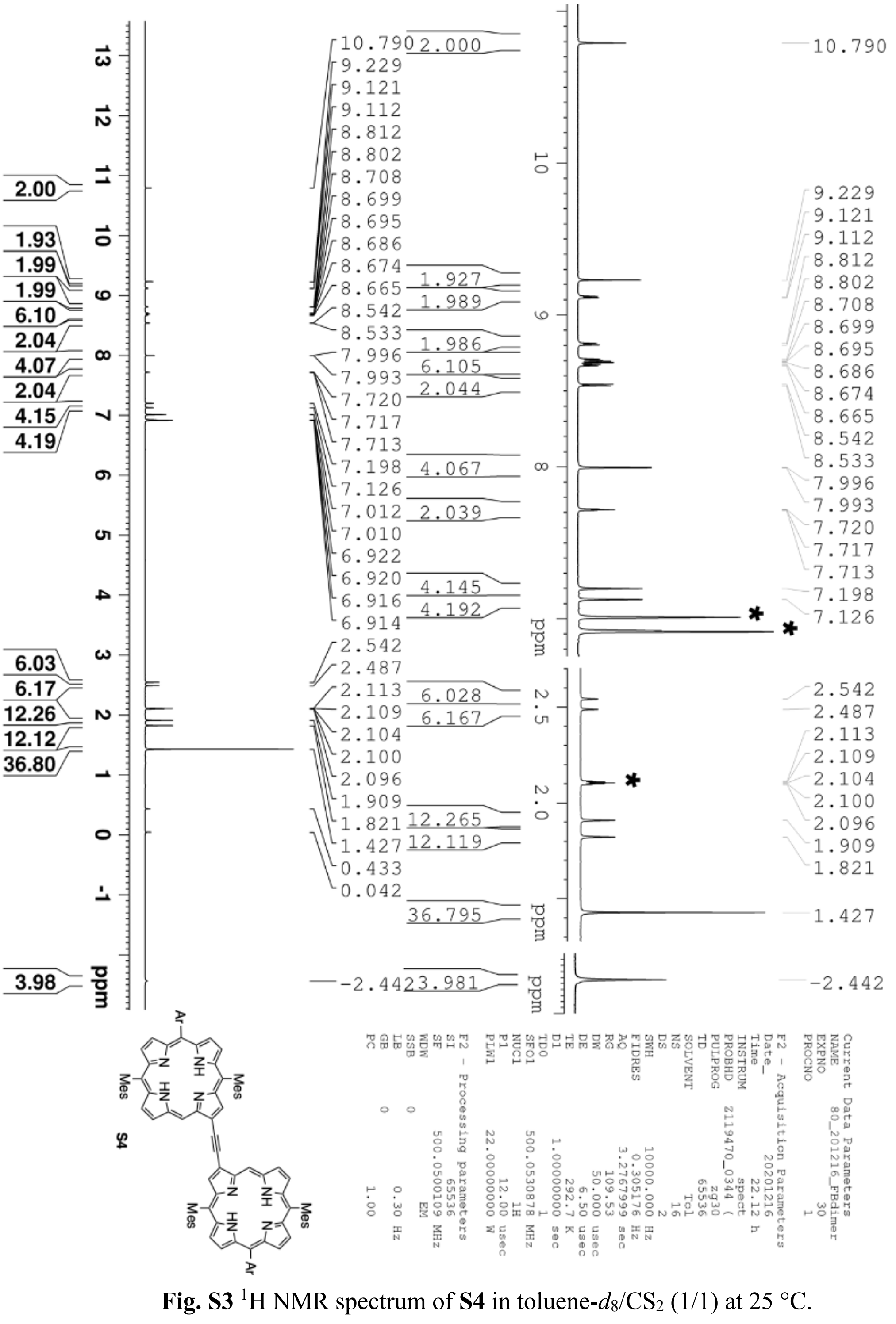

Fig. S3 ^{1}H NMR spectrum of **S4** in toluene-d_8/CS_2 (1/1) at 25 °C.

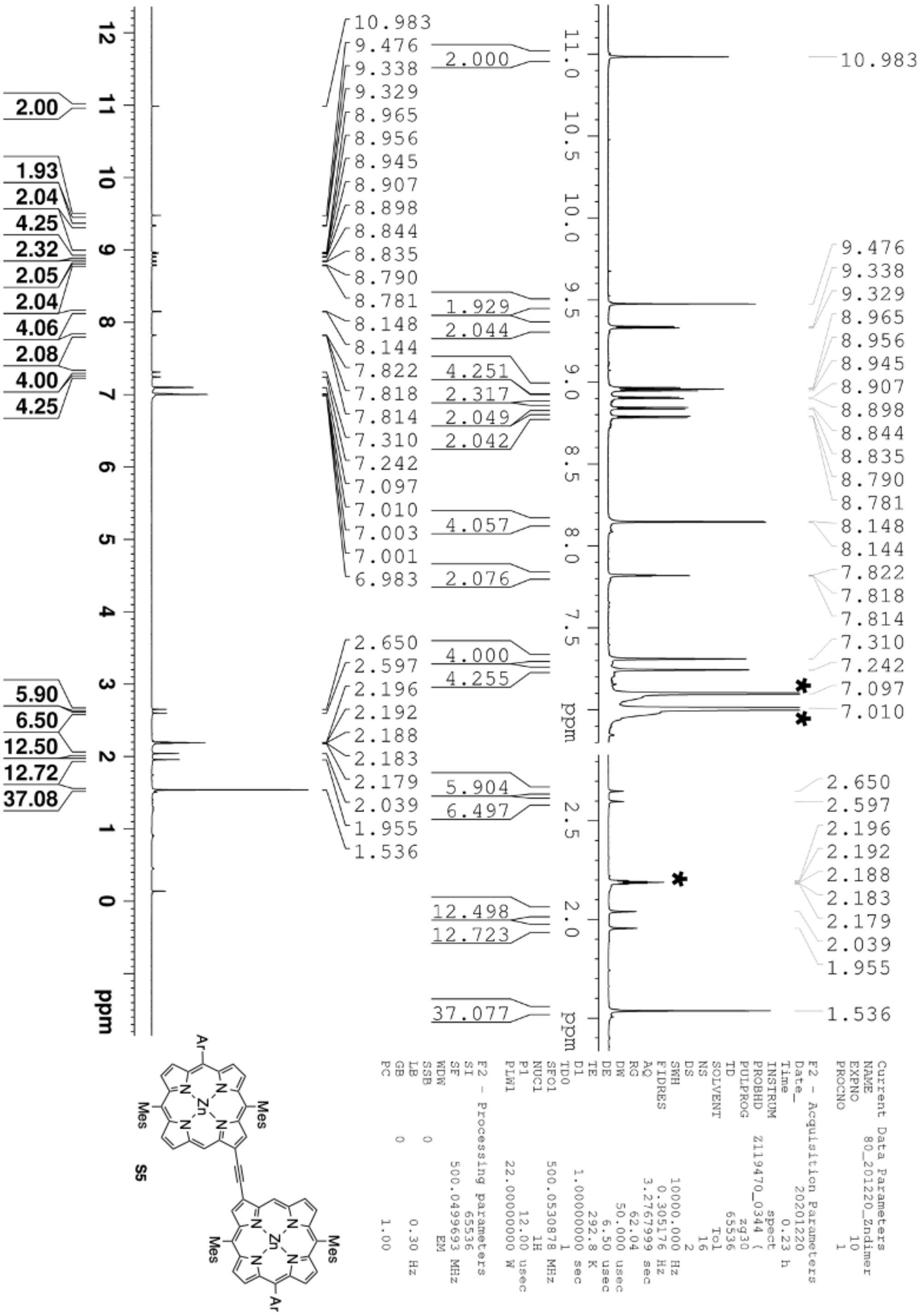

Fig. S4 ^{1}H NMR spectrum of **S5** in toluene-d_8/CS_2 (1/1) at 25 °C.

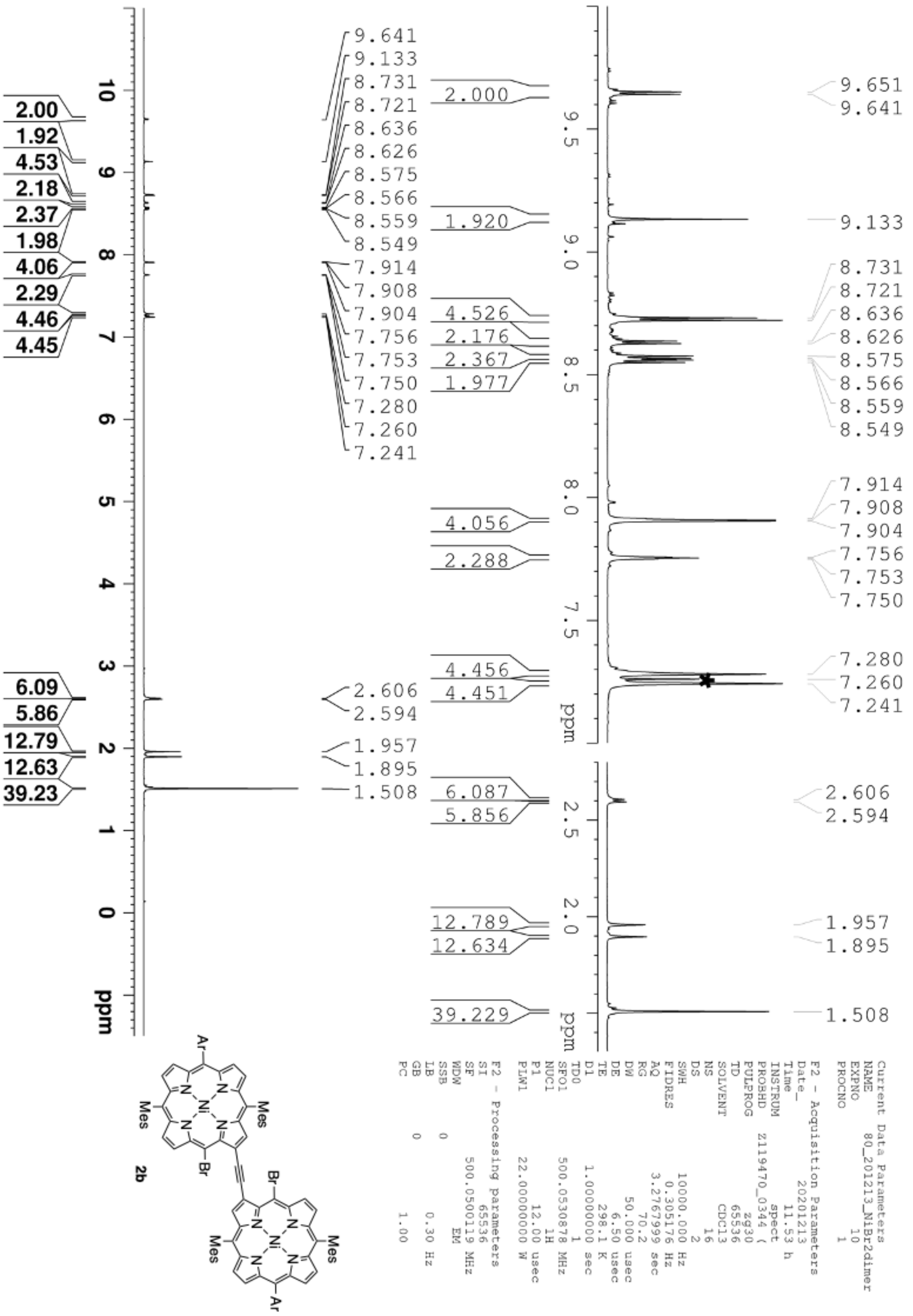

Fig. S5 ^{1}H NMR spectrum of **2b** in $CDCl_3$ at 25 °C.

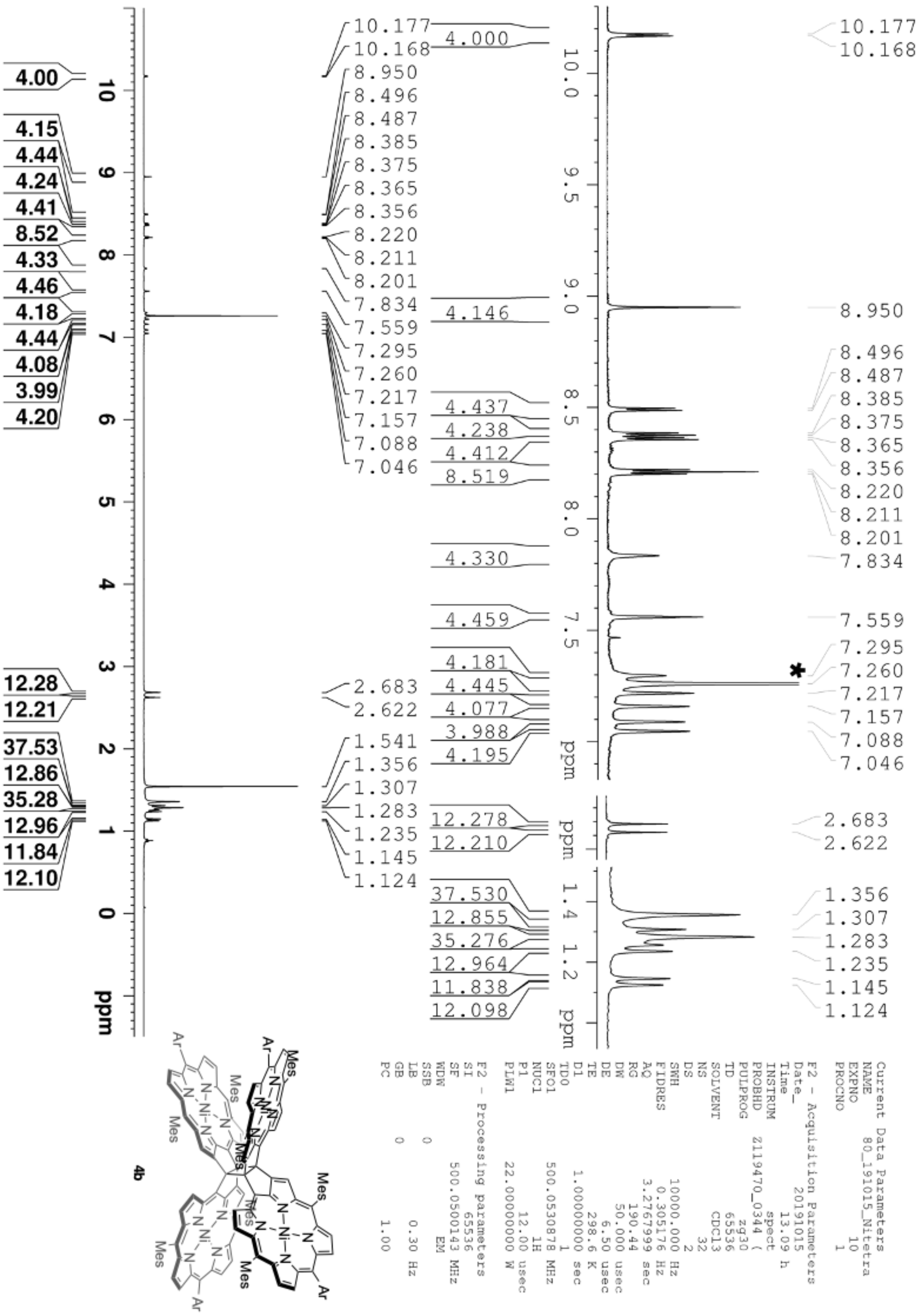

Fig. S6 ^{1}H NMR spectrum of **4b** in $CDCl_3$ at 25 °C.

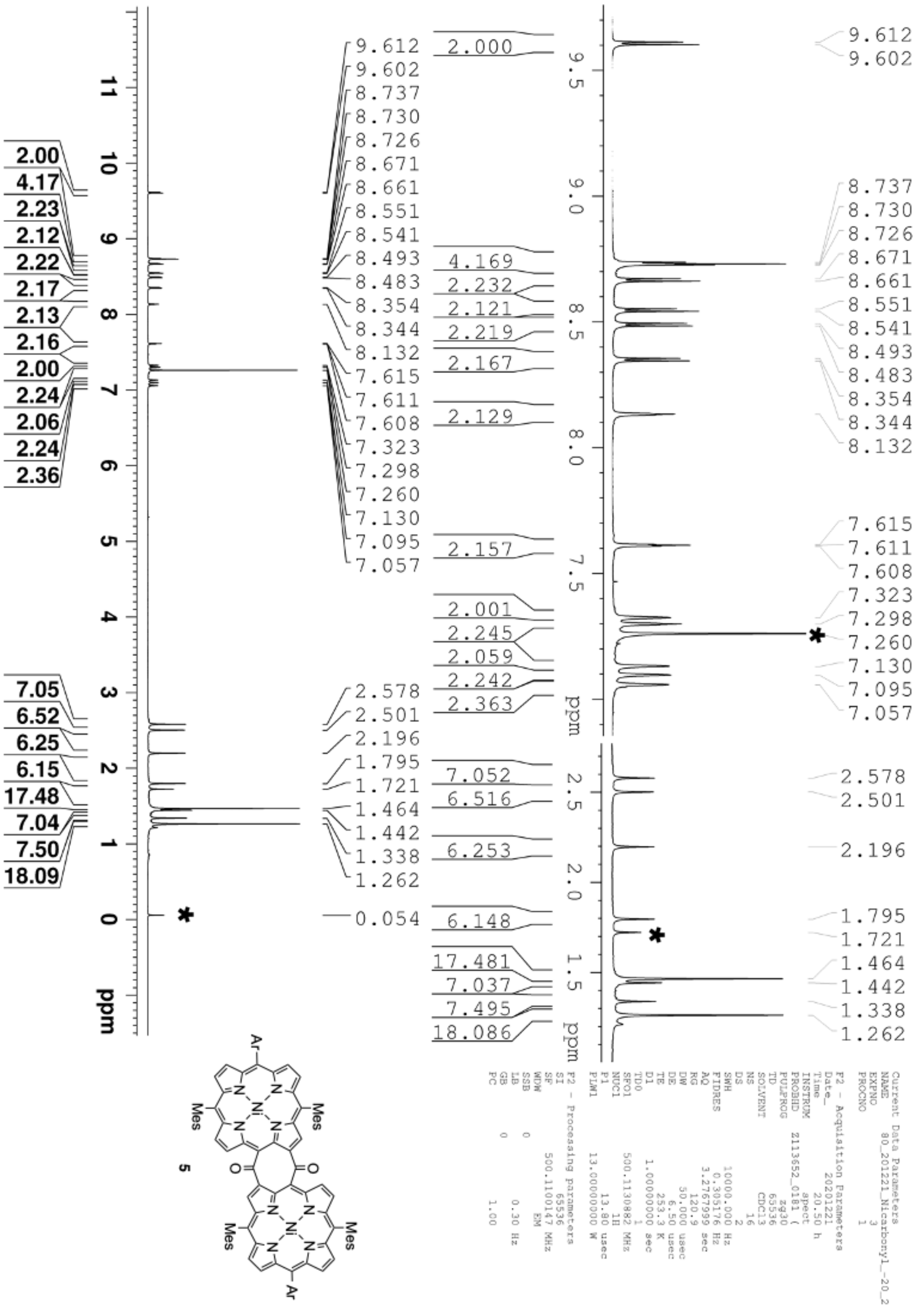

Fig. S7 ^{1}H NMR spectrum of **5** in $CDCl_3$ at –20 °C.

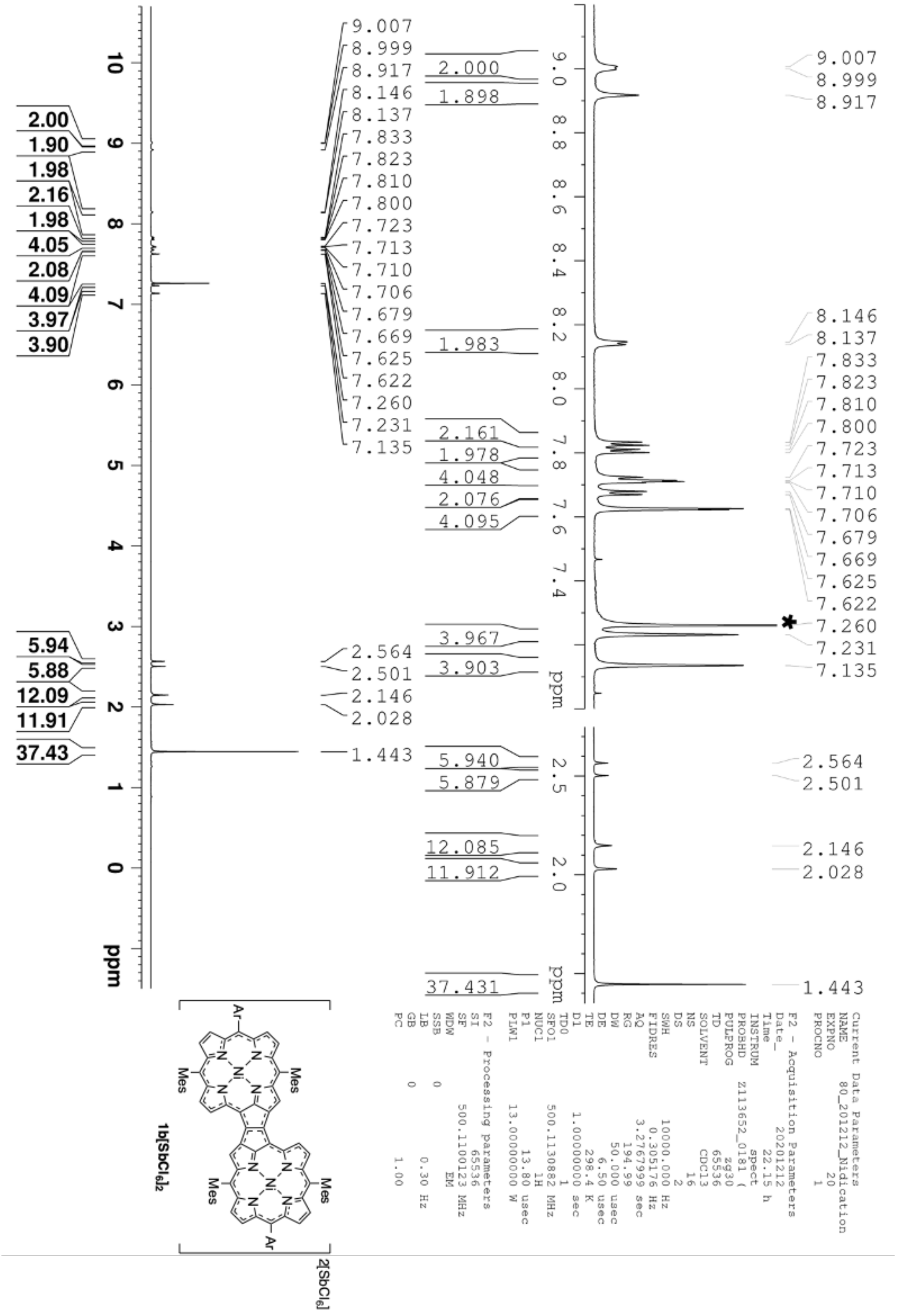

Fig. S8 ^{1}H NMR spectrum of **1b[SbCl$_6$]$_2$** in $CDCl_3$ at 25 °C.

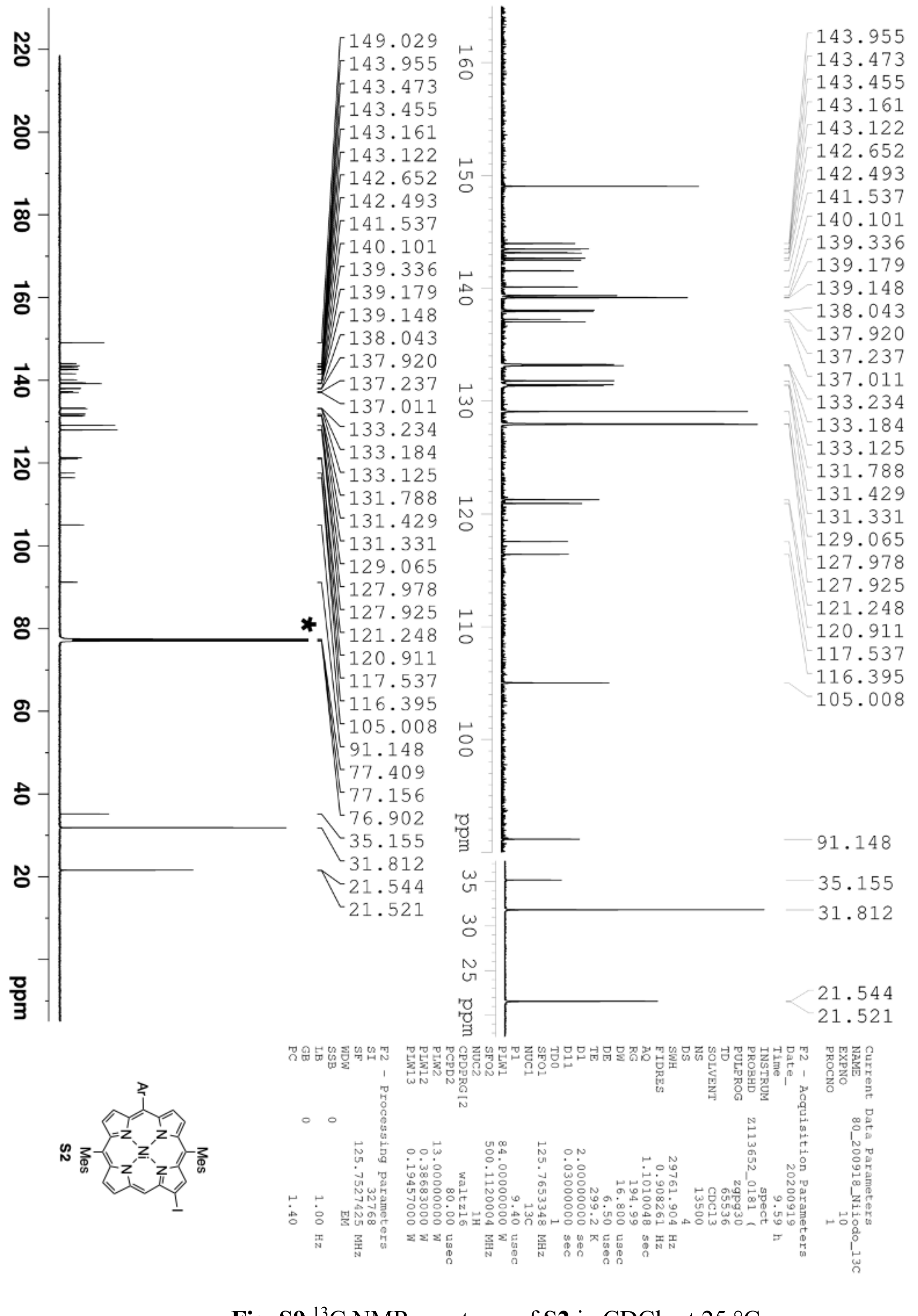

Fig. S9 ^{13}C NMR spectrum of **S2** in $CDCl_3$ at 25 °C.

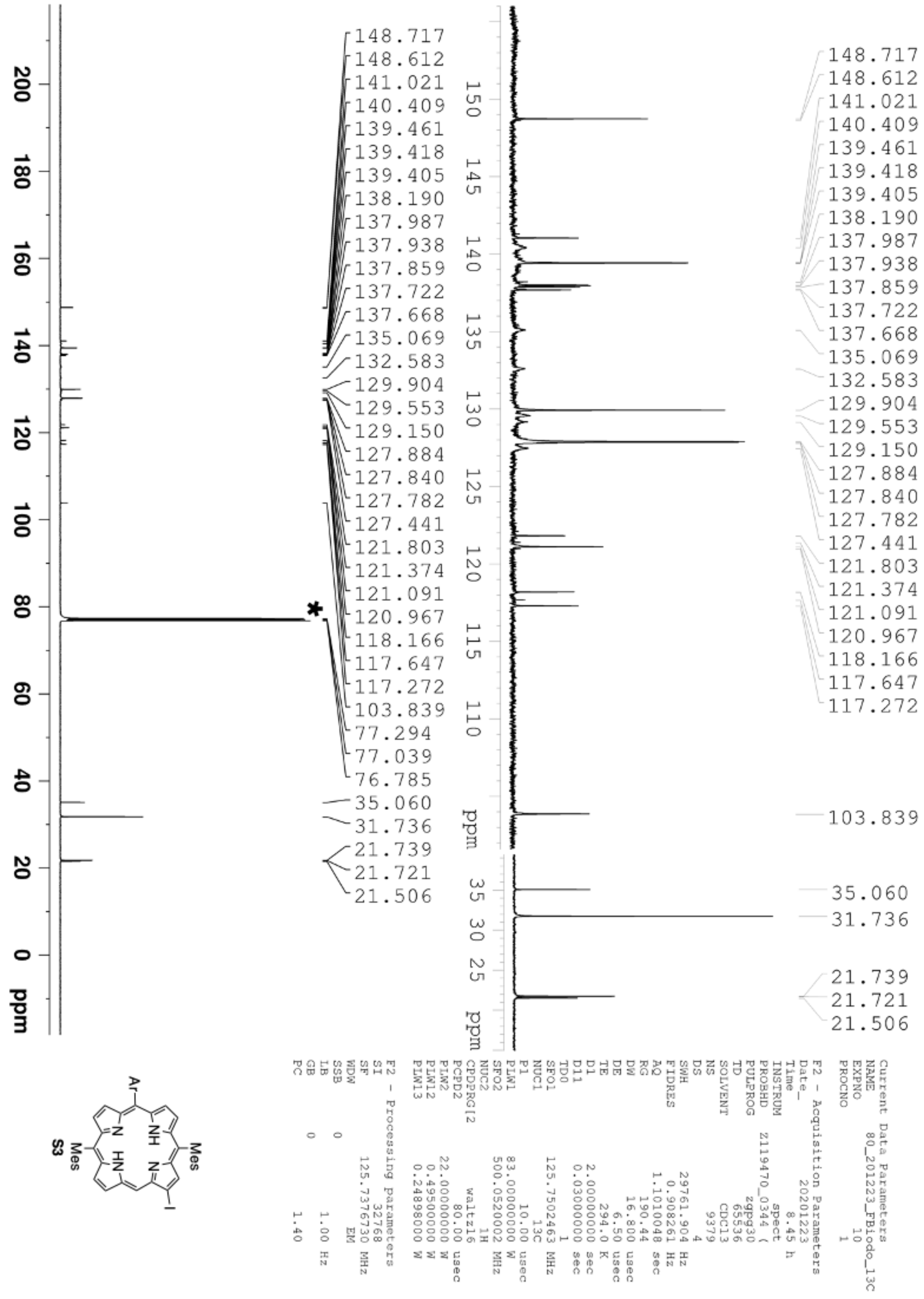

Fig. S10 ^{13}C NMR spectrum of **S3** in $CDCl_3$ at 25 °C.

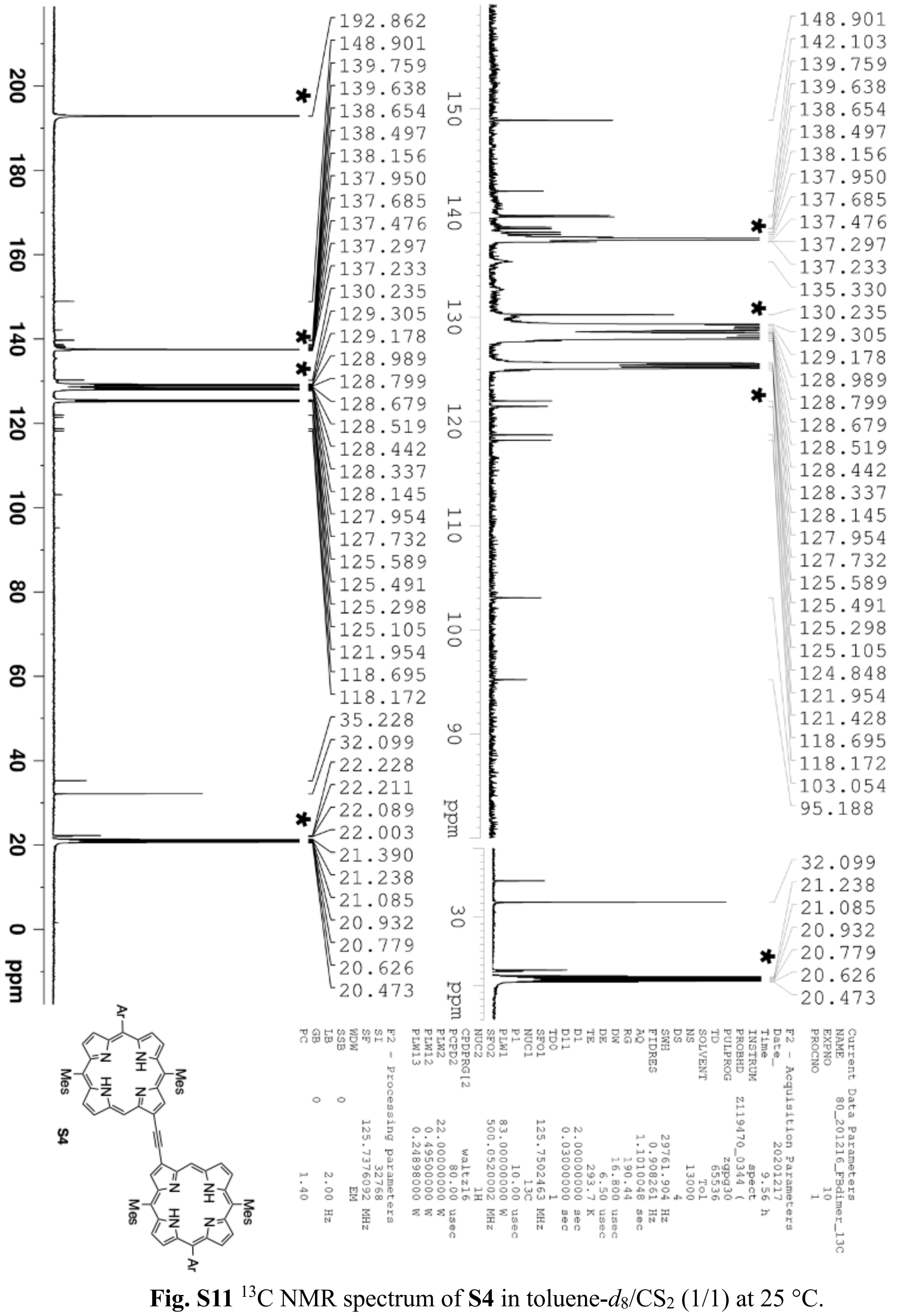

Fig. S11 ^{13}C NMR spectrum of **S4** in toluene-d_8/CS_2 (1/1) at 25 °C.

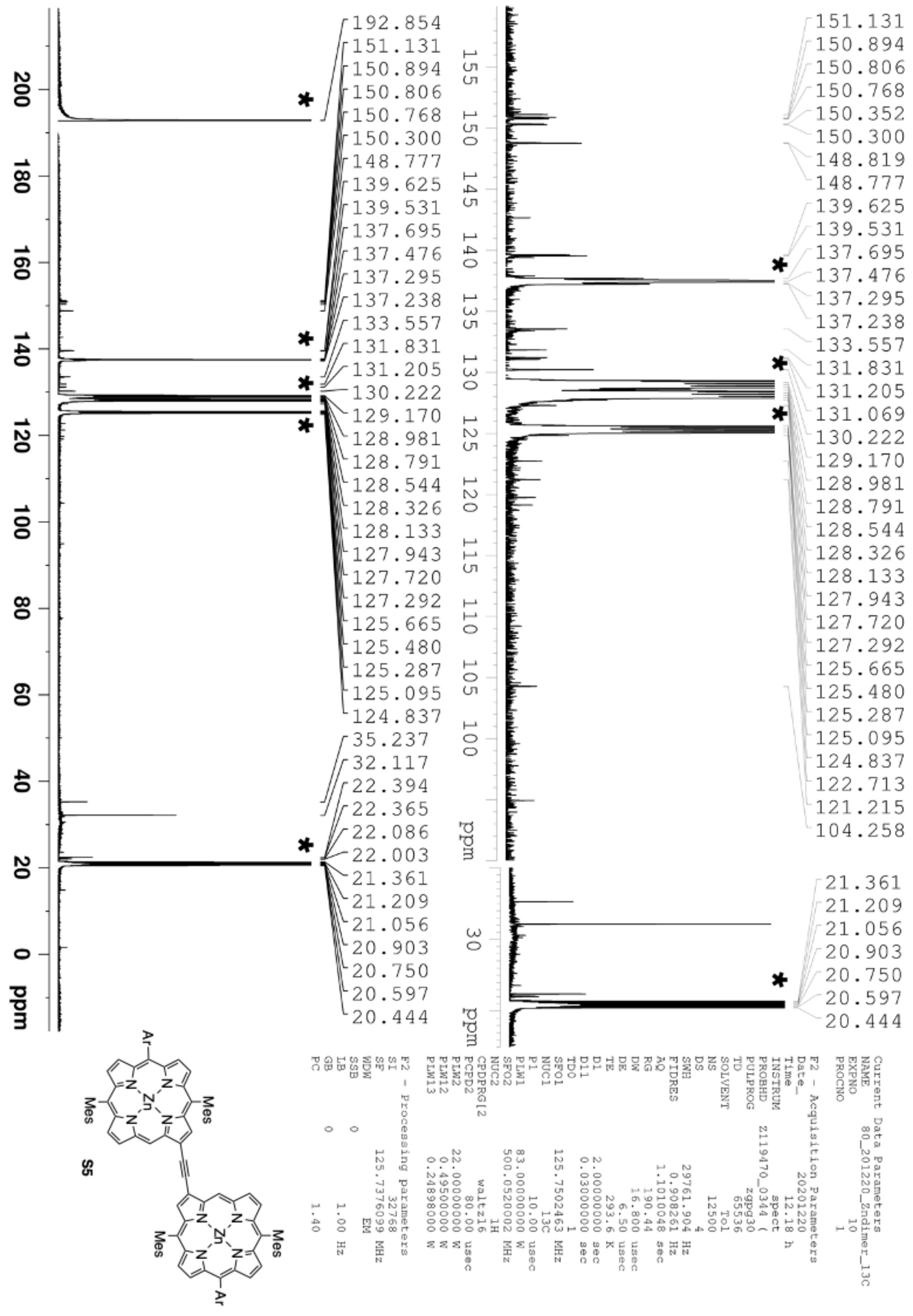

Fig. S12 ^{13}C NMR spectrum of **S5** in toluene-d_8/CS_2 (1/1) at 25 °C.

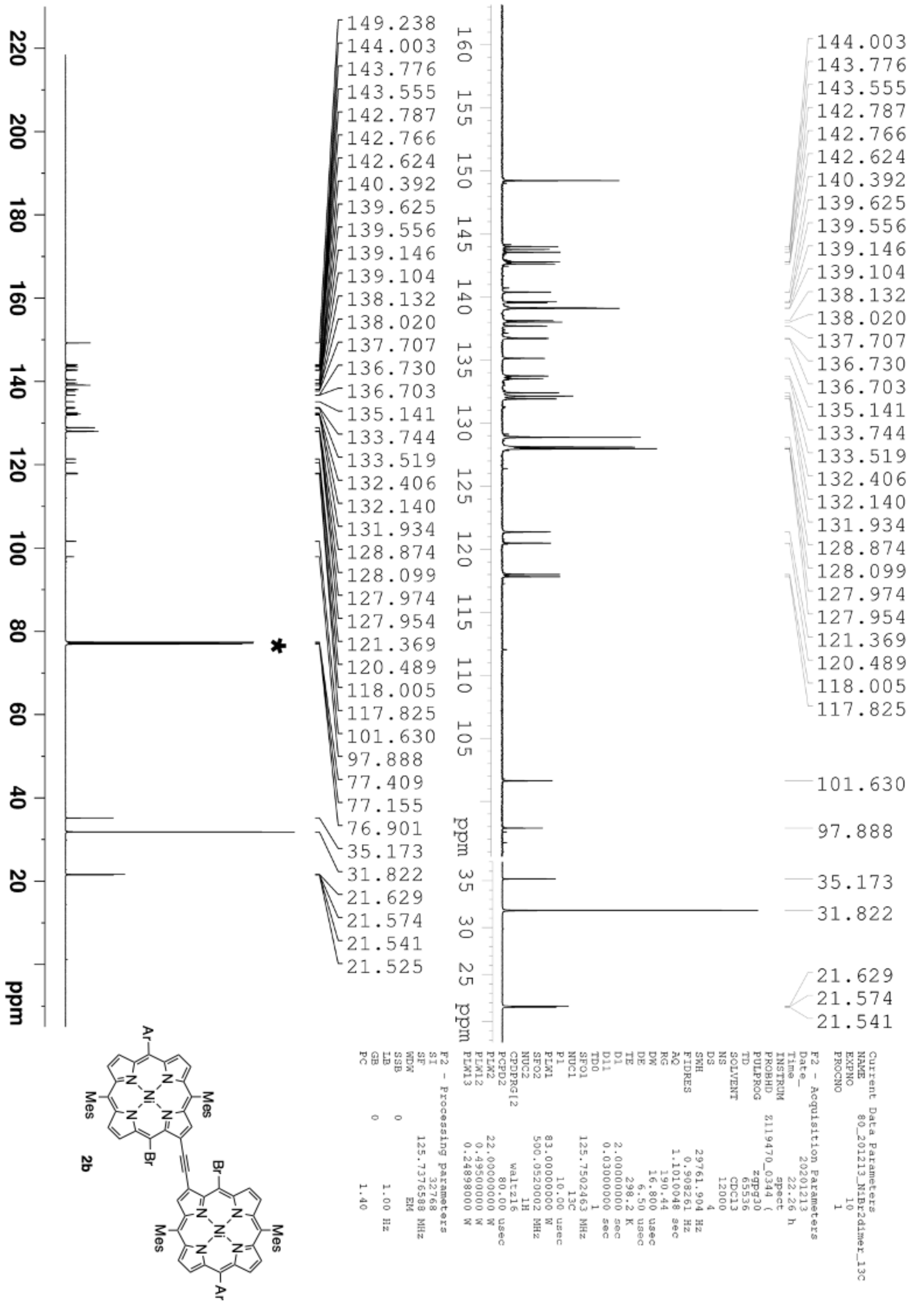

Fig. S13 ^{13}C NMR spectrum of **2b** in $CDCl_3$ at 25 °C.

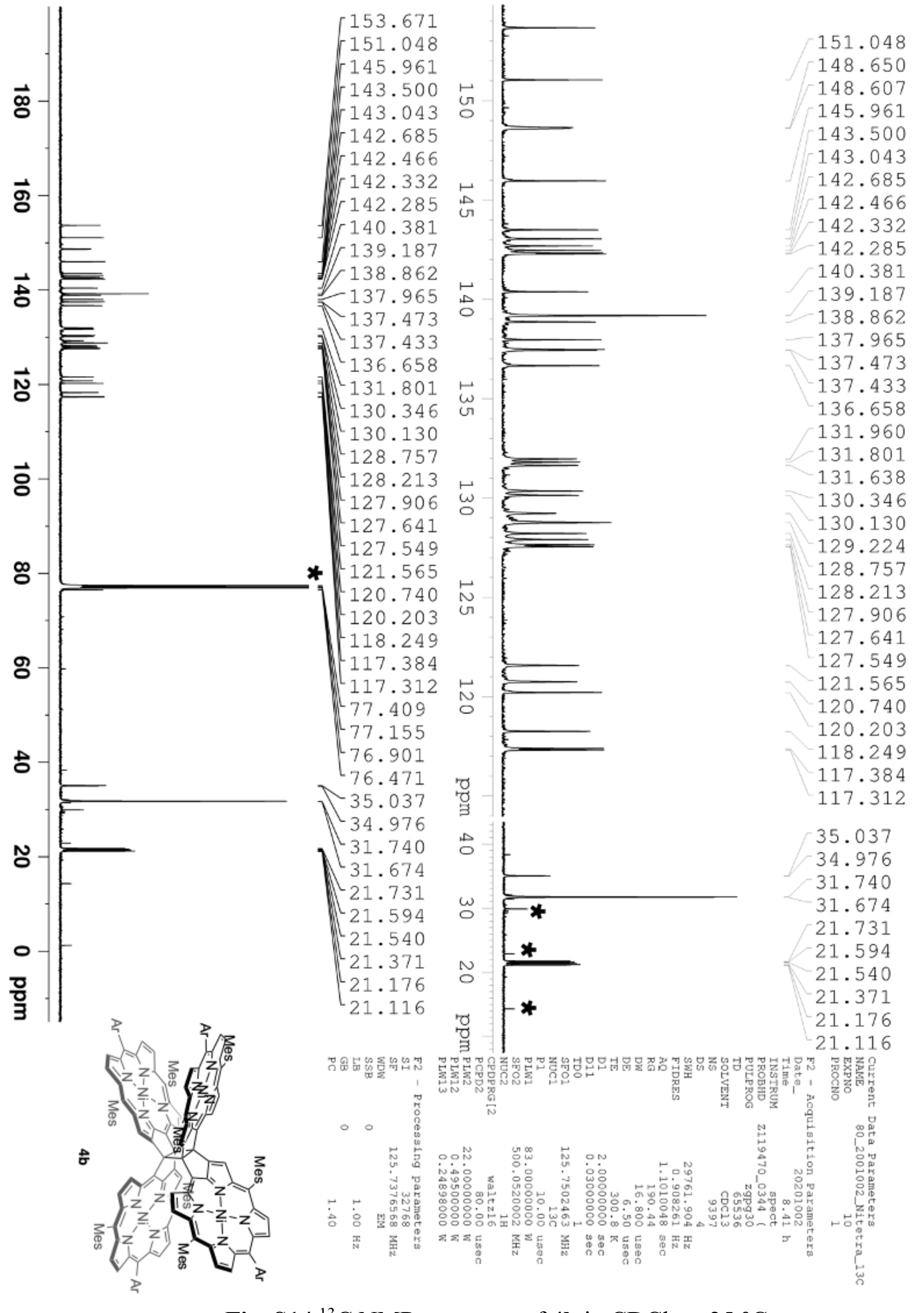

Fig. S14 ^{13}C NMR spectrum of **4b** in $CDCl_3$ at 25 °C.

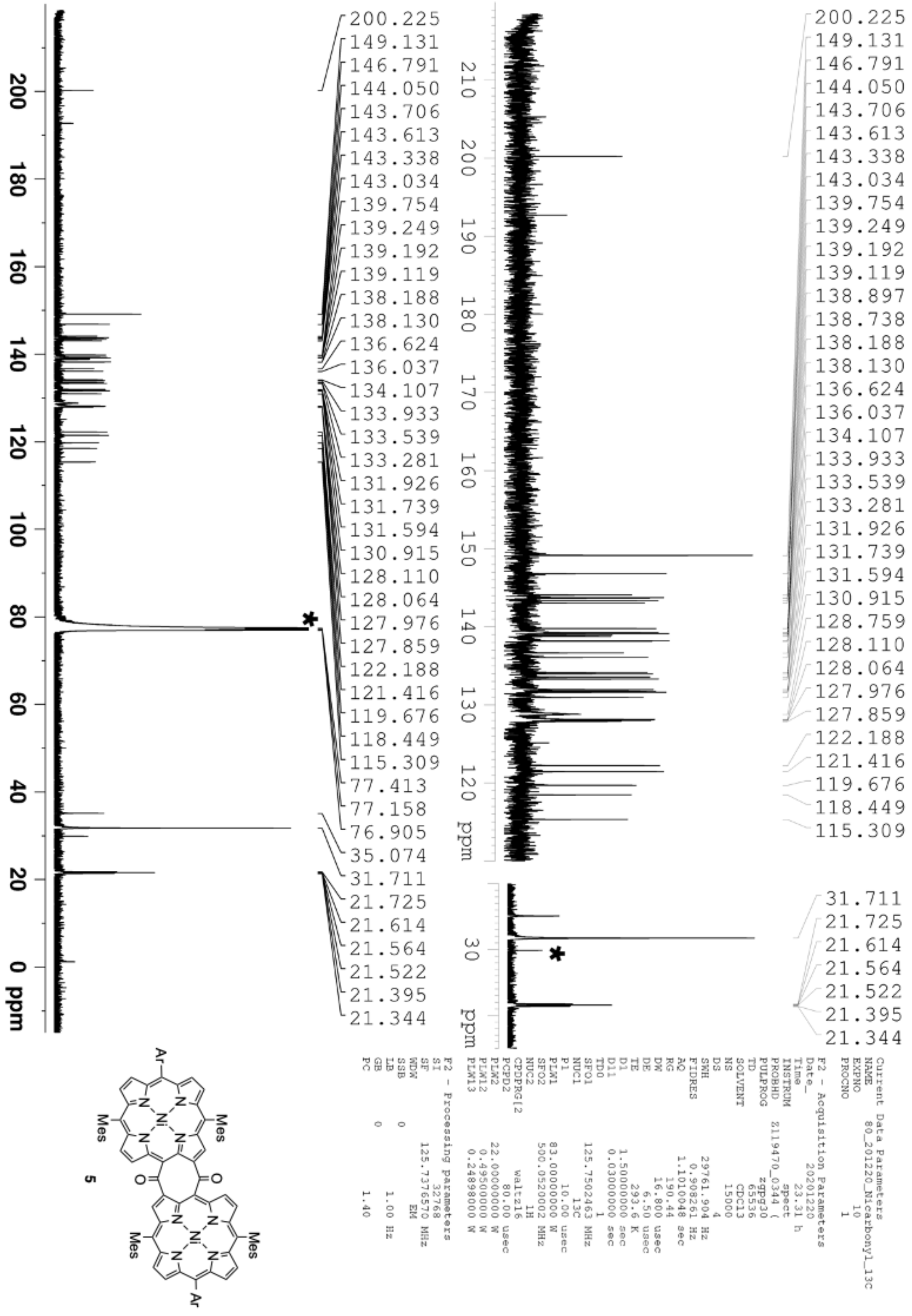

Fig. S15 ^{13}C NMR spectrum of **5** in $CDCl_3$ at 25 °C.

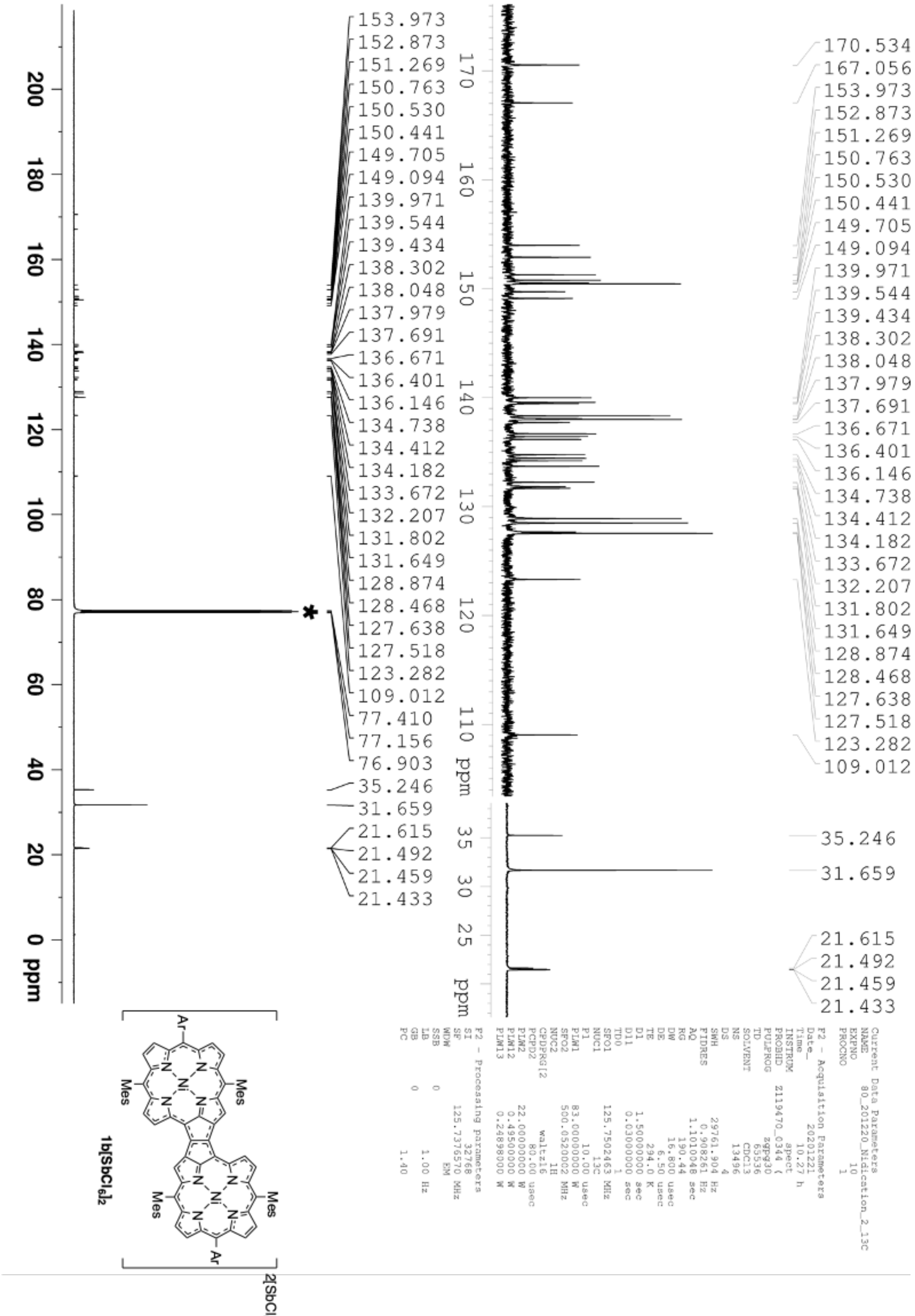

Fig. S16 ^{13}C NMR spectrum of **1b[SbCl$_6$]$_2$** in $CDCl_3$ at 25 °C.

4. Crystal data

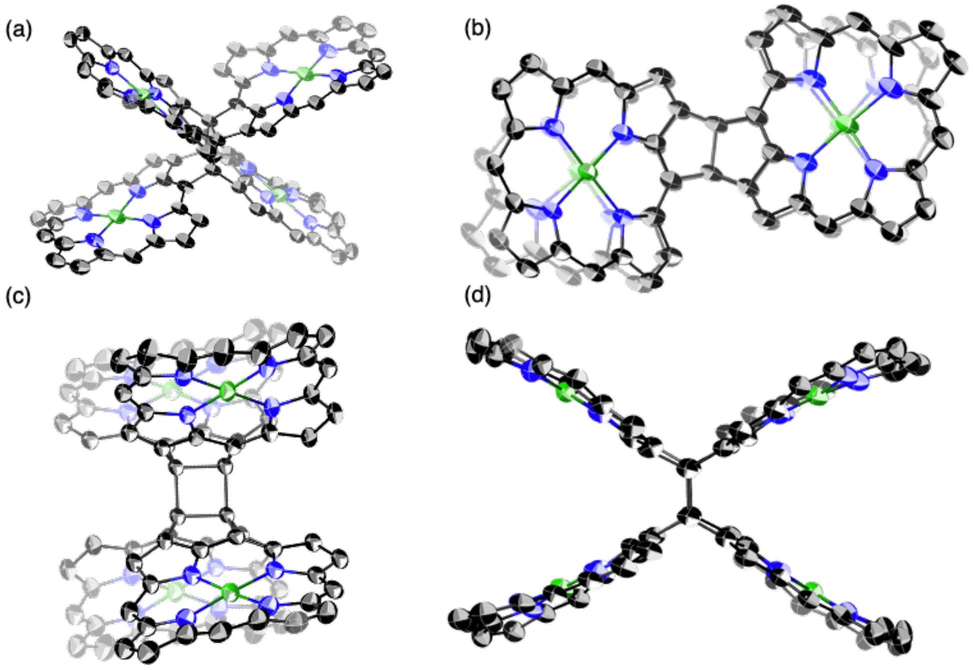

Fig. S17 X-ray crystal structure of **4b**. (a) General view, (b) top view, and (c,d) side views. Thermal ellipsoids are shown at the 50% probability level. Solvent molecules, aryl groups, and all hydrogen atoms are omitted for clarity.

Table S1 Crystallographic data of **4b** and **1b[SbCl$_6$]$_2$**.

compound	**4b**	**1b[SbCl$_6$]$_2$**
Formula	$C_{212}H_{200}N_{16}Ni_4$	$C_{106}H_{100}N_8Ni_2Cl_{12}Sb_2$
Formula weight	3206.82	2272.33
Crystal system	triclinic	triclinic
Space group	*P*-1 (No. 2)	*P*-1 (No. 2)
Crystal color	orange	dark green
Crystal description	plate	needle
a [Å]	21.0654(6)	9.2156(2)
b [Å]	22.4572(6)	20.0257(6)
c [Å]	25.0542(7)	21.5606(6)
α [°]	104.279(2)	114.680(3)
β [°]	98.423(2)	94.469(2)
γ [°]	111.649(2)	98.787(2)
V [Å^3]	10298.3(5)	3528.31(18)
Z	2	2
d_{calcd} [g cm^{-3}]	1.212	1.310
R_1 ($I > 2\sigma(I)$)	0.0926	0.1965
wR_2 (all data)	0.2855	0.5915
Goodness-of-fit	1.061	1.104
Temperature [K]	93	93
Solvent	toluene/MeCN	tetrachloroethane/hexane
CCDC No.	2055689	2055688

5. Temperature-dependent ^{1}H NMR spectra

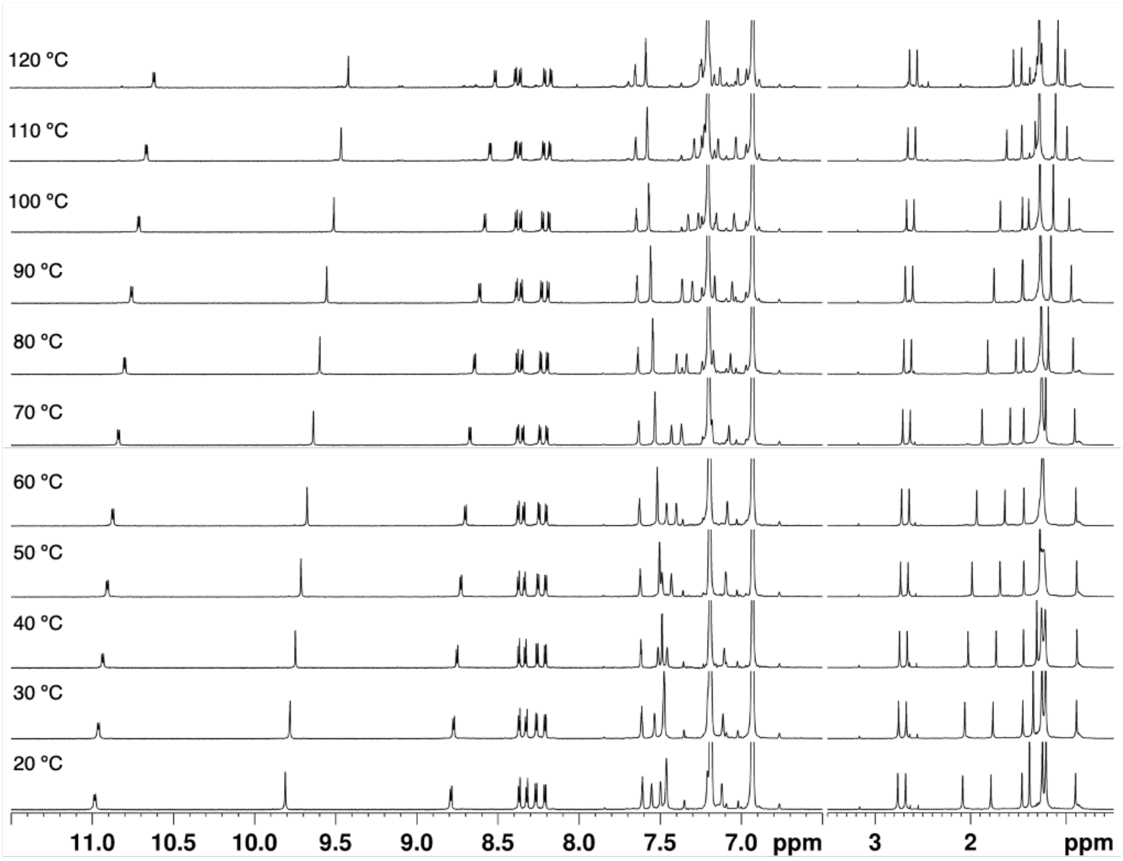

Fig. S18 Temperature dependent ^{1}H NMR spectra of **4b** in 1,2-dichlorobenzene-d_4.

6. Temperature-dependent UV-vis absorption spectra

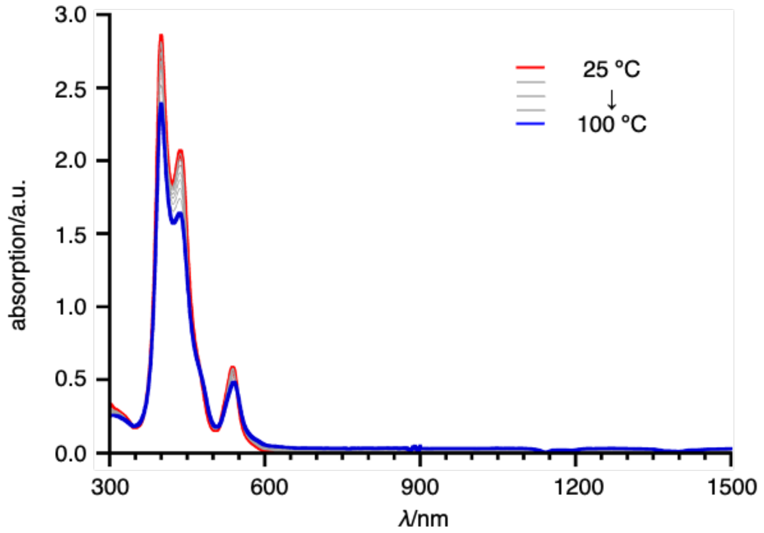

Fig. S19 Temperature dependent UV-vis absorption spectra of **4b** in toluene. λ = wavelength. [**4b**] = 6.7×10^{-6} M^{-1}.

7. Cyclic voltammograms

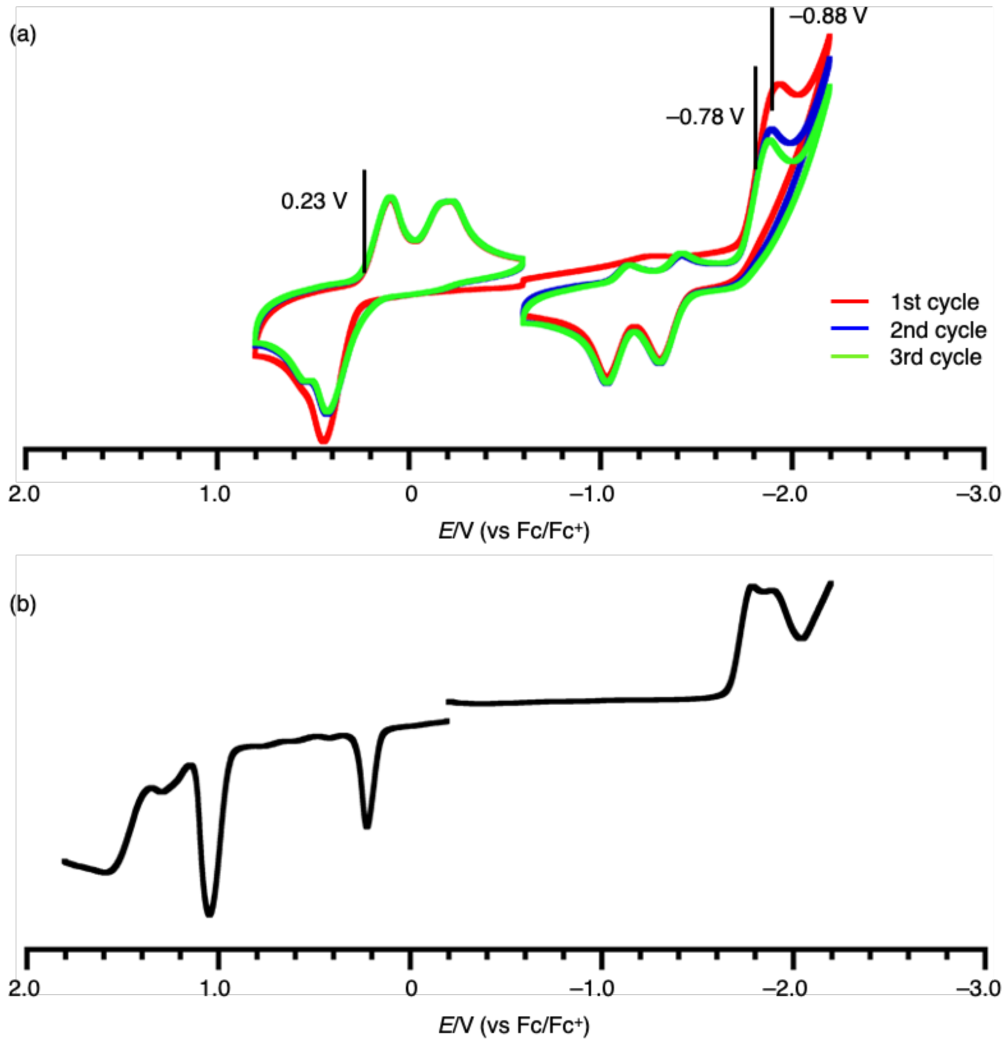

Fig. S20 (a) Cyclic voltammograms and (b) differential pulse voltammograms of **4b**. Solvent: CH_2Cl_2, electrolyte: 0.1 M Bu_4NPF_6, working electrode: glassy carbon, counter electrode: Pt, reference electrode: Ag/$AgNO_3$, scan rate: 0.1 V/s.

8. Spectroelectrochemistry

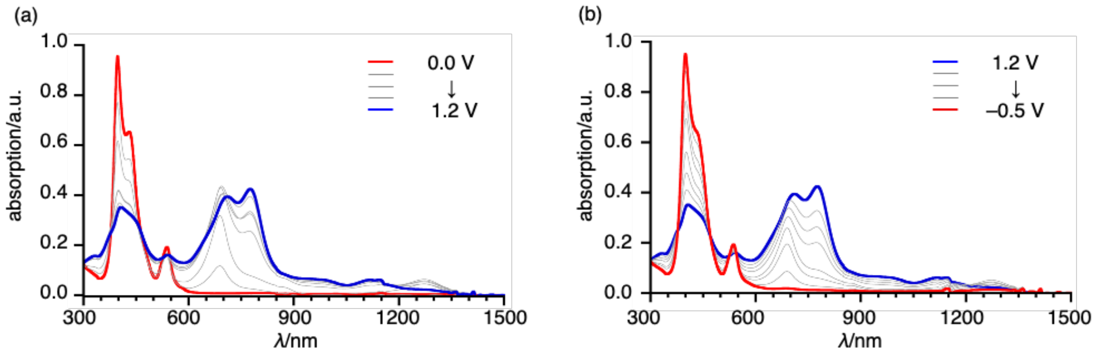

Fig. S21 Electronic absorption spectra of **4b** upon application of a voltage in CH_2Cl_2 using $[Bu_4N][PF_6]$ as the supporting electrolyte. (a) from 0 V to1.2 V and (b) from 1.2 V to –0.5 V (vs. Ag/Ag^+).

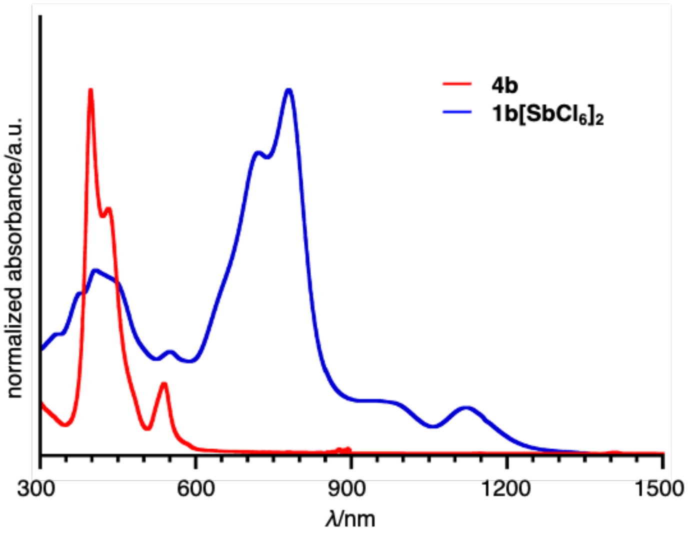

Fig. S22 UV/vis absorption spectrum of **1b[SbCl₆]₂** and **4b** in CH_2Cl_2. λ = wavelength.

9. UV/vis/NIR absorption spectra

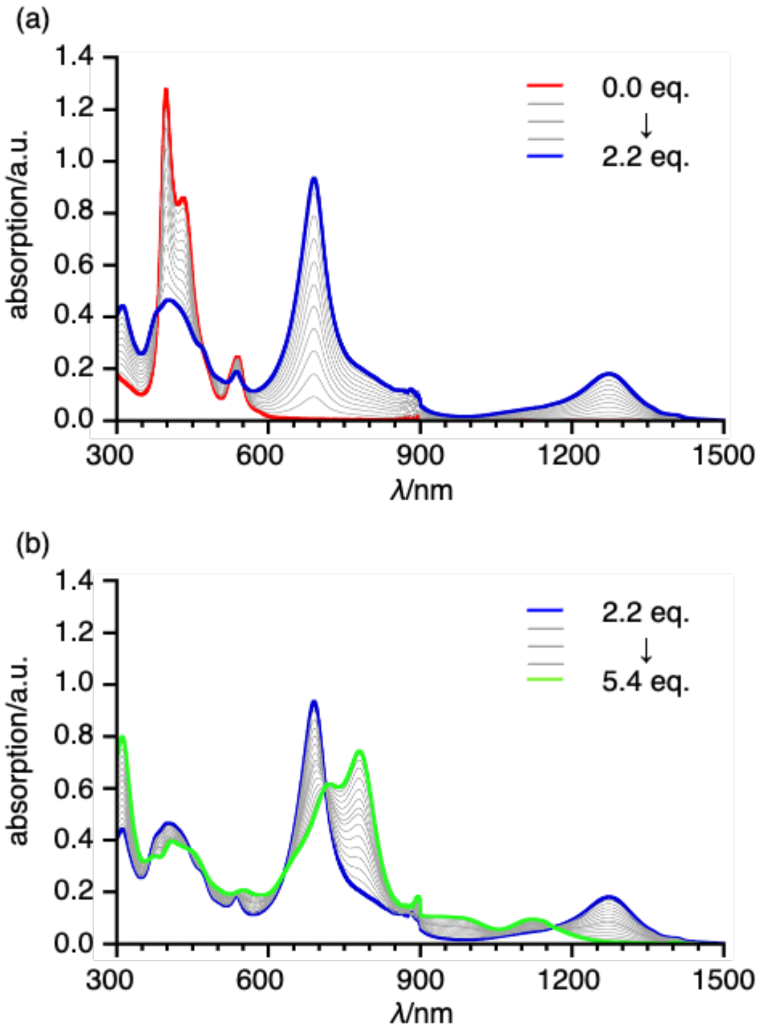

Fig. S23 2D plots for oxidative titration of **4b** with Magic Blue in CH_2Cl_2. (a) 0–2.2 equiv and (b) 2.2–5.4 equiv. λ = wavelength. [**4b**]$_0$ = 3.0×10^{-6} M^{-1}.

10. Photostability

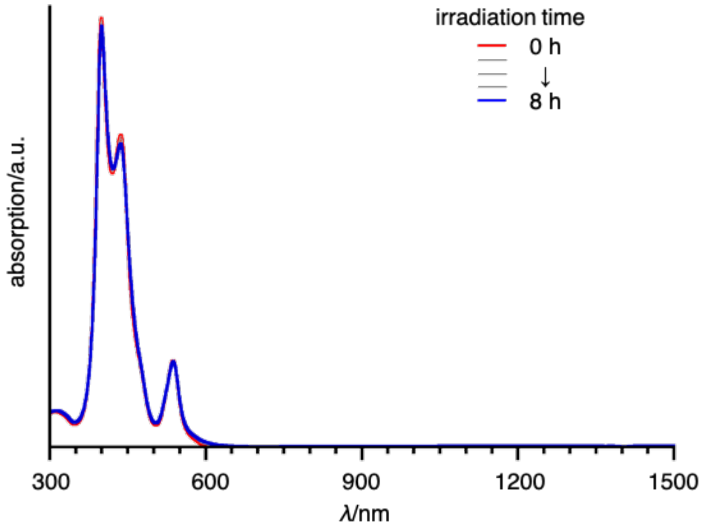

Fig. S24 Changes in the UV/vis absorption spectra of **4b** upon photoirradiation in toluene. For the photoirradiation, a high-pressure mercury lamp equipped with a sharp cut filter (λ > 380 nm) was employed. λ = wavelength.

11. DFT calculations

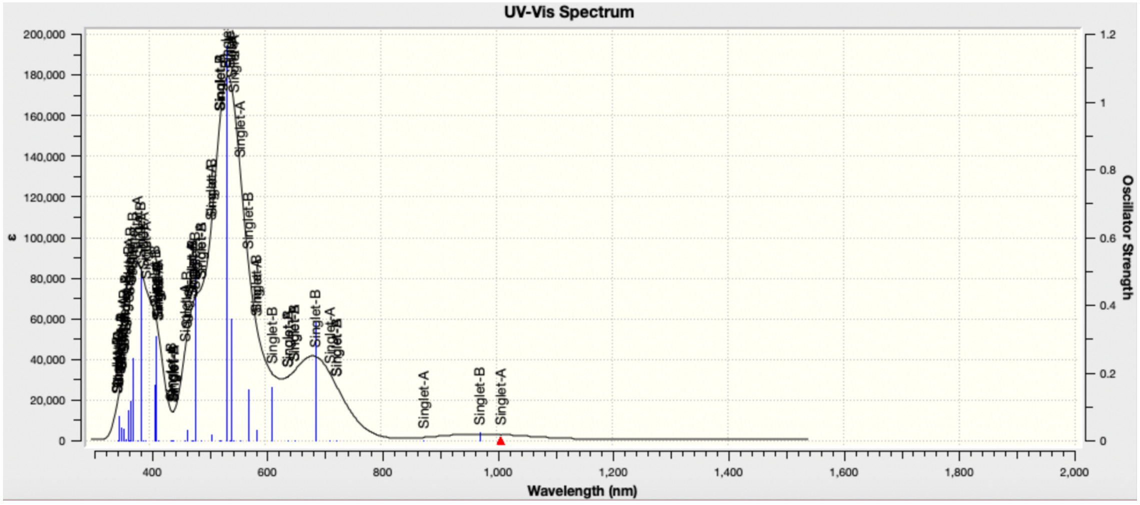

Fig. S25 Calculated absorption spectrum of **1b**$^{+}$.

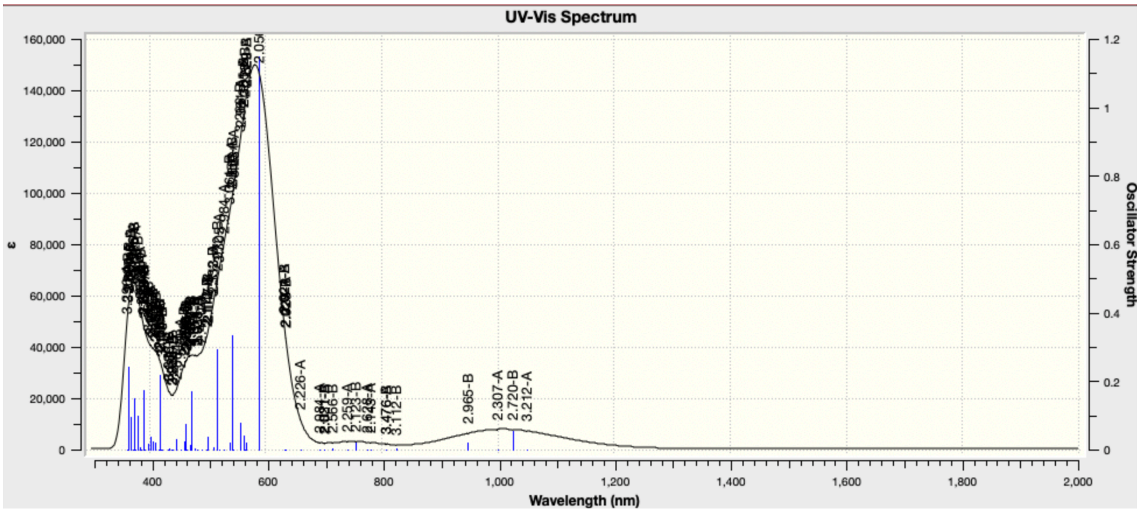

Fig. S26 Calculated absorption spectrum of **1b**$^{2+}$.

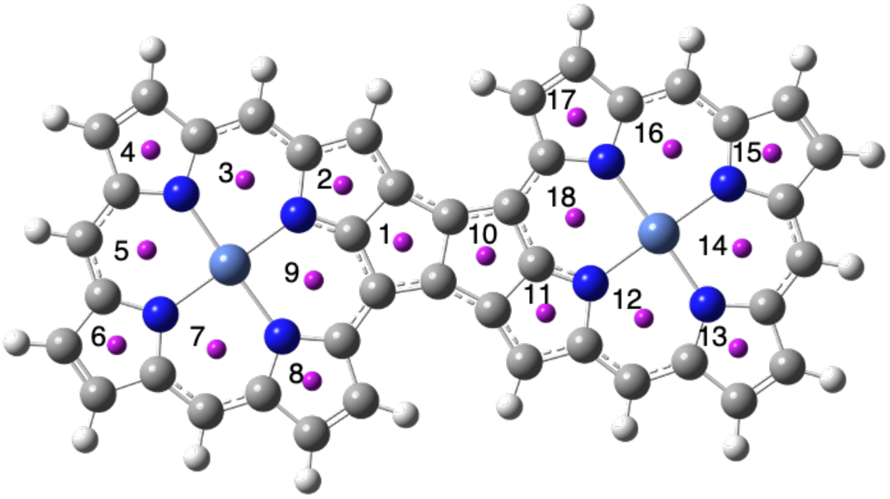

position	NICS(0)	position	NICS(0)
1	–5.4103	10	–5.4103
2	–8.7366	11	–8.7366
3	–8.4672	12	–8.4672
4	–1.3995	13	–1.3995
5	–9.9619	14	–9.9619
6	–1.4216	15	–1.4216
7	–10.0324	16	–10.0324
8	–1.8230	17	–1.8230
9	–9.7640	18	–9.7640

Fig. S27 NICS values of $\mathbf{1b}^{2+}$.

Table S2 Cartesian coordinate of **1b**$^{+}$.

C	-0.491975	2.099127	-0.000155
C	-1.466912	1.031778	-0.000158
C	-2.690692	1.664285	0.000002
H	-3.690823	1.251076	0.000056
C	-2.409063	3.078595	0.000063
C	-3.334408	4.097917	0.000209
C	-2.984262	5.445628	0.000235
C	-3.949132	6.511416	0.000316
H	-5.021857	6.369072	0.000396
C	-3.24518	7.675928	0.000299
H	-3.616376	8.692301	0.000355
C	-1.853077	7.312918	0.000143
C	-0.81429	8.233983	0.000062
C	0.525015	7.899702	-0.000012
C	1.59406	8.86741	-0.000049
H	1.451374	9.940045	-0.000049
C	2.754696	8.162757	-0.00009
H	3.772077	8.531239	-0.00013
C	2.389569	6.766949	-0.000057
C	3.318	5.733253	-0.000075
C	2.996237	4.391707	-0.000087
C	3.98526	3.338717	-0.000106
H	5.055068	3.502155	-0.000106
C	3.306153	2.1637	-0.000154
H	3.700677	1.157641	-0.000207
C	1.906464	2.500833	-0.000123
C	0.833906	1.615063	-0.000165
C	0.491975	-2.099127	-0.000155
C	1.466912	-1.031778	-0.000158
C	2.690692	-1.664285	0.000002
H	3.690823	-1.251076	0.000056
C	2.409063	-3.078595	0.000063
C	3.334408	-4.097917	0.000209
C	2.984262	-5.445628	0.000235
C	3.949132	-6.511416	0.000316
H	5.021857	-6.369072	0.000396
C	3.24518	-7.675928	0.000299
H	3.616376	-8.692301	0.000355
C	1.853077	-7.312918	0.000143
C	0.81429	-8.233983	0.000062
C	-0.525015	-7.899702	-0.000012
C	-1.59406	-8.86741	-0.000049
H	-1.451374	-9.940045	-0.000049
C	-2.754696	-8.162757	-0.00009
H	-3.772077	-8.531239	-0.00013
C	-2.389569	-6.766949	-0.000057
C	-3.318	-5.733253	-0.000075
C	-2.996237	-4.391707	-0.000087
C	-3.98526	-3.338717	-0.000106
H	-5.055068	-3.502155	-0.000106
C	-3.306153	-2.1637	-0.000154
H	-3.700677	-1.157641	-0.000207
C	-1.906464	-2.500833	-0.000123
C	-0.833906	-1.615063	-0.000165
C	-0.700842	-0.184049	-0.000169
C	0.700842	0.184049	-0.000169
N	-1.026511	3.31603	-0.000037
N	-1.697692	5.947079	0.000121
N	1.026511	6.613496	-0.000015
N	1.719952	3.869882	-0.000086
N	1.026511	-3.31603	-0.000037
N	1.697692	-5.947079	0.000121
N	-1.026511	-6.613496	-0.000015
N	-1.719952	-3.869882	-0.000086
Ni	0.001858	4.948732	-0.000001
Ni	-0.001858	-4.948732	-0.000001
H	-4.389892	3.846642	0.000294
H	-4.36921	-6.001734	-0.000091
H	1.073642	-9.287607	0.000078
H	4.389892	-3.846642	0.000294
H	4.36921	6.001734	-0.000091
H	-1.073642	9.287607	0.000078

Table S3 Cartesian coordinate of $\mathbf{1b}^{2+}$.

C	0.501032	-2.078905	-0.000066
C	1.477904	-1.006539	-0.000073
C	2.711087	-1.641838	0.000007
H	3.708565	-1.223942	0.000028
C	2.43262	-3.039637	0.000038
C	3.348162	-4.069017	0.000107
C	2.9933	-5.41532	0.000115
C	3.966938	-6.477901	0.000149
H	5.038845	-6.330709	0.000191
C	3.269009	-7.643249	0.000126
H	3.644056	-8.658155	0.000142
C	1.872351	-7.283489	0.000054
C	0.840581	-8.211411	0.000006
C	-0.502849	-7.883766	-0.000023
C	-1.565419	-8.859755	-0.000046
H	-1.417657	-9.931573	-0.000058
C	-2.729635	-8.160873	-0.000051
H	-3.744804	-8.535147	-0.000068
C	-2.36863	-6.763724	-0.000024
C	-3.303256	-5.733122	-0.000022
C	-2.9933	-4.389742	-0.000028
C	-3.990254	-3.345008	-0.000042
H	-5.059003	-3.514323	-0.000039
C	-3.318069	-2.165433	-0.000073
H	-3.722401	-1.163781	-0.000104
C	-1.918196	-2.49678	-0.000059
C	-0.845149	-1.598571	-0.000079
C	-0.501032	2.078905	-0.000066
C	-1.477904	1.006539	-0.000073
C	-2.711087	1.641838	0.000007
H	-3.708565	1.223942	0.000028
C	-2.43262	3.039637	0.000038
C	-3.348162	4.069017	0.000107
C	-2.9933	5.41532	0.000115
C	-3.966938	6.477901	0.000149
H	-5.038845	6.330709	0.000191
C	-3.269009	7.643249	0.000126
H	-3.644056	8.658155	0.000142
C	-1.872351	7.283489	0.000054
C	-0.840581	8.211411	0.000006
C	0.502849	7.883766	-0.000023
C	1.565419	8.859755	-0.000046
H	1.417657	9.931573	-0.000058
C	2.729635	8.160873	-0.000051
H	3.744804	8.535147	-0.000068
C	2.36863	6.763724	-0.000024
C	3.303256	5.733122	-0.000022
C	2.9933	4.389742	-0.000028
C	3.990254	3.345008	-0.000042
H	5.059003	3.514323	-0.000039
C	3.318069	2.165433	-0.000073
H	3.722401	1.163781	-0.000104
C	1.918196	2.49678	-0.000059
C	0.845149	1.598571	-0.000079
C	0.727154	0.194204	-0.000086
C	-0.727154	-0.194204	-0.000086
N	1.029283	-3.280641	-0.000005
N	1.708633	-5.920139	0.000057
N	-1.007839	-6.600948	-0.000009
N	-1.718328	-3.857535	-0.000034
N	-1.029283	3.280641	-0.000005
N	-1.708633	5.920139	0.000057
N	1.007839	6.600948	-0.000009
N	1.718328	3.857535	-0.000034
Ni	0.005796	-4.931267	0.000005
Ni	-0.005796	4.931267	0.000005
H	4.405255	-3.824517	0.00015
H	4.352594	6.0093	-0.000024
H	-1.106225	9.263388	0.000002
H	-4.405255	3.824517	0.00015
H	-4.352594	-6.0093	-0.000024
H	1.106225	-9.263388	0.000002

12. References

1. Gaussian 09, Revision E.01, M. J. Frisch, G. W. Trucks, H. B. Schlegel, G. E. Scuseria, M. A. Robb, J. R. Cheeseman, G. Scalmani, V. Barone, B. Mennucci, G. A. Petersson, H. Nakatsuji, M. Caricato, X. Li, H. P. Hratchian, A. F. Izmaylov, J. Bloino, G. Zheng, J. L. Sonnenberg, M. Hada, M. Ehara, K. Toyota, R. Fukuda, J. Hasegawa, M. Ishida, T. Nakajima, Y. Honda, O. Kitao, H. Nakai, T. Vreven, J. A. Montgomery, Jr., J. E. Peralta, F. Ogliaro, M. Bearpark, J. J. Heyd, E. Brothers, K. N. Kudin, V. N. Staroverov, T. Keith, R. Kobayashi, J. Normand, K. Raghavachari, A. Rendell, J. C. Burant, S. S. Iyengar, J. Tomasi, M. Cossi, N. Rega, J. M. Millam, M. Klene, J. E. Knox, J. B. Cross, V. Bakken, C. Adamo, J. Jaramillo, R. Gomperts, R. E. Stratmann, O. Yazyev, A. J. Austin, R. Cammi, C. Pomelli, J. W. Ochterski, R. L. Martin, K. Morokuma, V. G. Zakrzewski, G. A. Voth, P. Salvador, J. J. Dannenberg, S. Dapprich, A. D. Daniels, O. Farkas, J. B. Foresman, J. V. Ortiz, J. Cioslowski and D. J. Fox, Gaussian, Inc., Wallingford CT, 2013.
2. (a) K. Fujimoto, H. Yorimitsu and A. Osuka, *Org. Lett.,* 2014, **16**, 972–975. (b) K. Fujimoto and A. Osuka, *Chem. Sci.,* 2017, **8**, 8231–8239.
